# Supplementary material for: Salmonella Typhimurium exploits host polyamines for assembly of the type 3 secretion machinery
Source: PLoS Biol. 2024 Aug 5;22(8):e3002731. doi: 10.1371/journal.pbio.3002731 (PMC11299824; doi:10.1371/journal.pbio.3002731)
Supplement: S1 Table — (PDF) [file pbio.3002731.s016.pdf]

S1 Table. Nomenclature of type 3 secretion components

Salmonella enterica		**Sct common nomenclature**[a]	**Function**
T3SS-1	T3SS-2		
InvG	SsaC	SctC	Outer rings of needle complex
PrgH	SsaD	SctD	Inner rings of needle complex
PrgK	SsaJ	SctJ	Inner rings of needle complex
SpaP	SsaR	SctR	Export apparatus
SpaQ	SsaS	SctS	Export apparatus
SpaR	SsaT	SctT	Export apparatus
SpaS	SsaU	SctU	Export apparatus; subsrate switching
InvA	SsaV	SctV	Export apparatus
OrgA	-	SctK	Cytoplasmic sorting platform
SpaO	SsaQ	SctQ	Cytoplasmic sorting platform
OrgB	SsaK	SctL	Links ATPase to sorting platform
InvC	SsaN	SctN	ATPase
InvI	SsaO	SctO	Cytoplasmic sorting platform
PrgI	SsaG	SctF	Needle filament component (needle subunit)
PrgJ	SsaI	SctI	Inner rod component
InvJ	SsaP	SctP	Inner rod assembly; substrate switching
SipD	-	-	Tip complex; translocase deployment
SipB	SseC	-	Effector translocase
SipC	SseD	-	Effector translocase
-	SseB		Effector tip protein
InvH	-	-	Pilotin
InvE	SsaL	SctW	Controls translocase secretion

[a] Previously proposed common nomenclature [1, 2]

Supplementary references

1. Hueck CJ. Type III protein secretion systems in bacterial pathogens of animals and

plants. Microbiol Mol Biol Rev. 1998;62(2):379-433. doi: 10.1128/mmbr.62.2.379-433.1998. PubMed PMID: 9618447; PubMed Central PMCID: PMCPMC98920.

2. Wagner S, Diepold A. A Unified Nomenclature for Injectisome-Type Type III Secretion Systems. Curr Top Microbiol Immunol. 2020;427:1-10. doi: 10.1007/82_2020_210. PubMed PMID: 32415388.
