# Supplementary material for: Salmonella Typhimurium exploits host polyamines for assembly of the type 3 secretion machinery
Source: PLoS Biol. 2024 Aug 5;22(8):e3002731. doi: 10.1371/journal.pbio.3002731 (PMC11299824; doi:10.1371/journal.pbio.3002731)
Supplement: S2 Table — (PDF) [file pbio.3002731.s017.pdf]

S2 Table. Bacterial strains used in this study

Strain	Genotype	Reference
Salmonella enterica **serovar Typhimurium (*S*Tm)**		
SL1344	Wild-type *S*Tm, *hisG*	Hoiseth & Stocker [3]
T643	SL1344 Δ*speABCEDF*::*kan*	This study
T567	SL1344 Δ*potAB*::*cat* Δ*potFGHI*	This study
T573	SL1344 Δ*potAB* Δ*potFGHI*	This study
T781	SL1344 Δ*speABCEDF* Δ*potAB*::*cat*	This study
T808	SL1344 Δ*speABCEDF* Δ*potFGHI*::*cat*	This study
T799	SL1344 Δ*speABCEDF* Δ*potAB* Δ*potFGHI*::*cat*	This study
T805	SL1344 Δ*speABCEDF* Δ*potAB* Δ*potFGHI*	This study
TM1582	SL1344 Δ*ssrB*::*cat*	This study
S373	T799 harboring pMW118	This study
S374	T799 harboring pMW-*potAB*	This study
S375	T799 harboring pMW-*potFGHI*	This study
S633	T573 harboring pMW118	This study
S623	T573 harboring pMW-*potAB*	This study
S634	T573 harboring pMW-*potFGHI*	This study
T273	SL1344 Δ*fliGHI*	Nakamura et al. [4]
T523	SL1344 Δ*fliGHI*::*cat*	Nakamura et al. [4]
S399	SL1344 Δ*speABCEDF* Δ*potAB* Δ*potFGHI* Δ*fliGHI*::*cat*	This study
T249	SL1344 Δ*invG* Δ*ssaV*::*cat*	Fujimoto et al. [5]
S395	SL1344 Δ*speABCEDF* Δ*potAB* Δ*potFGHI* Δ*invG*::*kan* Δ*ssaV*::*cat*	This study
T267	SL1344 *sicA*::*lacZ*	Hoshino et al. [6]
T594	SL1344 Δ*hilA*::*kan* *sicA*::*lacZ*	Hoshino et al. [6]
T841	T799 *sicA*::*lacZ*	This study
S636	SL1344 *prgH*::*lacZ*	This study

S637	SL1344 Δ*hilA*::*kan prgH*::*lacZ*	This study
S638	T799 *prgH*::*lacZ*	This study
T246	SL1344 Δ*invG*::*kan*	Fujimoto et al. [5]
S447	SL1344 harboring pACHS-SopD-CyaA-2HA	This study
S448	T246 harboring pACHS-SopD-CyaA-2HA	This study
S449	T805 harboring pACHS-SopD-CyaA-2HA	This study
T145	SL1344 Δ*ssaV*::*cat*	Goto et al. [7]
TM131	SL1344 Δ*ssaV*	Miki et al. [8]
TM2317	SL1344 harboring pACPJ-SseJ-CyaA-2HA	This study
TM2318	TM131 harboring pACPJ-SseJ-CyaA-2HA	This study
S437	T805 harboring pACPJ-SseJ-CyaA-2HA	This study
S622	SL1344 Δ*prgI*::*kan*	This study
S625	S622 harboring pACHS-SopD-CyaA-2HA	This study
S552	SL1344 Δ*invJ*::*kan*	This study
S561	SL1344 Δ*invJ*::*kan* Δ*fliGHI*::*cat*	This study
S562	SL1344 Δ*invJ*::*kan* Δ*fliGHI*::*cat* Δ*speABCEDF* Δ*potAB* Δ*potFGHI*	This study
S564	S561 harboring pBAD-*hilD*	This study
S565	S562 harboring pBAD-*hilD*	This study
S540	SL1344 Δ*fliGHI*::*cat* Δ*prgH*::*kan*	This study
S541	SL1344 Δ*fliGHI*::*cat* Δ*prgH*::*kan* Δ*speABCEDF* Δ*potAB* Δ*potFGHI*	This study
S544	S540 pMAL-*prgH*	This study
S545	S541 pMAL-*prgH*	This study
TM3108	SL1344 harboring pACHS-SopD-2HA	This study
S549	T246 harboring pACHS-SopD-2HA	This study
S550	T805 harboring pACHS-SopD-2HA	This study
TM198	SL1344 Δ*ssaL*::*kan*	Miki et al. [8]
TM203	SL1344 Δ*ssaL*::*kan spiA(C133S)*	Miki et al. [8]

S464	SL1344 Δ*ssaL*::*kan* Δ*speABCEDF* Δ*potAB* Δ*potFGHI*::*cat*	This study
S466	TM198 harboring pMW-SseJ-HA (pTM22)	This study
S472	TM203 harboring pMW-SseJ-HA (pTM22)	This study
S468	S464 harboring pMW-SseJ-HA (pTM22)	This study
S558	SL1344 harboring pACHS-InvJ-2HA	This study
S559	T246 harboring pACHS-InvJ-2HA	This study
S560	T805 harboring pACHS-InvJ-2HA	This study
S635	SL1344 Δ*prgI*::*kan* Δ*fliGHI*::*cat*	This study
S622	SL1344 Δ*prgI*::*kan*	This study
S669	S622 harboring pACHS-SopD-2HA	This study
S643	SL1344 harboring pACHS-PrgH-2HA	This study
S644	SL1344 Δ*hilA*::*kan* harboring pACHS-PrgH-2HA	This study
S645	T805 harboring pACHS-PrgH-2HA	This study
S629	SL1344 harboring pACHS-PrgHI-2HA	This study
S630	SL1344 Δ*hilA*::*kan* harboring pACHS-PrgHI-2HA	This study
S631	T805 harboring pACHS-PrgHI-2HA	This study
TM2885	SL1344 harboring pACHS-SsaG-2HA	This study
TM2886	SL1344 Δ*ssrB* harboring pACHS-SsaG-2HA	This study
S551	T805 harboring pACHS-SsaG-2HA	This study

Supplementary references

3. Hoiseth SK, Stocker BA. Aromatic-dependent *Salmonella typhimurium* are non-virulent and effective as live vaccines. Nature. 1981;291(5812):238-9. doi: 10.1038/291238a0. PubMed PMID: 7015147.

4. Nakamura N, Hoshino Y, Shiga T, Haneda T, Okada N, Miki T. A Peptidoglycan Amidase Activator Impacts *Salmonella enterica* Serovar Typhimurium Gut Infection. Infection and immunity. 2020;88(6). Epub 20200520. doi: 10.1128/iai.00187-20. PubMed PMID: 32284369; PubMed Central PMCID: PMCPMC7240094.

5. Fujimoto M, Goto R, Haneda T, Okada N, Miki T. *Salmonella enterica* Serovar

Typhimurium CpxRA Two-Component System Contributes to Gut Colonization in *Salmonella*-Induced Colitis. Infection and immunity. 2018;86(7). Epub 20180621. doi: 10.1128/iai.00280-18. PubMed PMID: 29685984; PubMed Central PMCID: PMCPMC6013652.

6. Hoshino Y, Sakamoto T, Sudo N, Ito M, Haneda T, Okada N, Miki T. Fatty Acid Homeostasis Tunes Flagellar Motility by Activating Phase 2 Flagellin Expression, Contributing to *Salmonella* Gut Colonization. Infection and immunity. 2022;90(7):e0018422. Epub 20220602. doi: 10.1128/iai.00184-22. PubMed PMID: 35652649; PubMed Central PMCID: PMCPMC9302153.

7. Goto R, Miki T, Nakamura N, Fujimoto M, Okada N. *Salmonella* Typhimurium PagP- and UgtL-dependent resistance to antimicrobial peptides contributes to the gut colonization. PloS one. 2017;12(12):e0190095. Epub 20171221. doi: 10.1371/journal.pone.0190095. PubMed PMID: 29267354; PubMed Central PMCID: PMCPMC5739500.

8. Miki T, Shibagaki Y, Danbara H, Okada N. Functional characterization of SsaE, a novel chaperone protein of the type III secretion system encoded by *Salmonella* pathogenicity island 2. Journal of bacteriology. 2009;191(22):6843-54. Epub 20090918. doi: 10.1128/jb.00863-09. PubMed PMID: 19767440; PubMed Central PMCID: PMCPMC2772465.
