# Supplementary material for: Salmonella Typhimurium exploits host polyamines for assembly of the type 3 secretion machinery
Source: PLoS Biol. 2024 Aug 5;22(8):e3002731. doi: 10.1371/journal.pbio.3002731 (PMC11299824; doi:10.1371/journal.pbio.3002731)
Supplement: S3 Table — (PDF) [file pbio.3002731.s018.pdf]

S3 Table. Oligonucleotide primers used in this study

Name	Sequence (5' to 3')
speA-red-FW	TTCGCCTTCGTCAGCAGGCGAACAGGGTGTACTACGCTCCGTGTAGGCTGGAGCTGCTTC
speA-red-RV	TTTAAATAATTAAATCGACCCACAAGTGGAATACTCTTAACATATGAATATCCTCCTTAG
speB-red-FW	GGGAGGGTTTTTTATATTGACTAAGAAGAGGTTTTTGCCGTGTAGGCTGGAGCTGCTTC
speB-red-RV	CTGTAGGCCGGATAAGCGCAGCGCCATCCGGCAAAACAACCATATGAATATCCTCCTTAG
speC-red-FW	GTCTGGGGTTACTCTACTCCCGAAACGGATATTTGCGCTTGTGTAGGCTGGAGCTGCTTC
speC-red-RV	CGGATAAGGTGTAAATGCCTTATCCGGCCTGCAGGCAACGCATATGAATATCCTCCTTAG
speE-red-FW	GCCCTTTTTTTACGGGTGTTAACAATGGAGGTATCAGCCAGTGTAGGCTGGAGCTGCTTC
speD-red-RV	AGCCGGAGTGACAGCGCCGTCAGGCGCTGCCAGTGGTGAACATATGAATATCCTCCTTAG
speF-red-FW	AATTGAGGGCCTGCTATTACCTGAAATAAAGAGATGAAAAGTGTAGGCTGGAGCTGCTTC
speF-red-RV	TTTTATTGGATTTAGGTTTACTCATAATTTTTCCTCCTTCCATATGAATATCCTCCTTAG
potA-red-FW	ACCACAAACCCCGCAGCGGTCAGCCATCCGTTGCGTTTACGTGTAGGCTGGAGCTGCTTC
potB-red-RV	TTCATGCCTGGATGCGTGTGGATTTGTTAGCGTTGCGCAACATATGAATATCCTCCTTAG
potF-red-FW	ATGGGTTCATTTGTTAACGGATTTCAGAAGGAAAGCGATGGTGTAGGCTGGAGCTGCTTC
potI-red-RV	GCGCGGCAACGCATTGCCATAGTGGAAGATTTTAGTGGCTCATATGAATATCCTCCTTAG

SL prgI-red-FW	CCCAAGCCCACTTTAATTTAACGTAAATAAGGAAGTCATTGTGTAGGCTGGAGCTGCTTC
SL prgI-red-RV	GGACAATAGTTGCAATCGACATAATCCACCTTATAACTGACATATGAATATCCTCCTTAG
speA-EcoRI-FW	AAAGAATTCCGCTATGTCTGACGACATGTC
speA-SalI-RV	AAAGTCGACCGACCCACAAGTGGAATACTC
speB-KpnI-FW	CCCGGTACCAGTAAACTTTGAGCCGTAAAC
speB-HindIII-RV	TTTAAGCTTAGGCCGGATAAGCGCAGCGCC
speC-SacI-FW	AAAGAGCTCGAGCTGGTATCCAGTTTGACC
speC-HindIII-RV	TTTAAGCTTATGCCTTATCCGGCCTGCAGG
speE-KpnI-FW	AAAGGTACCATAATGACCGTGCATCCACGG
speD-SalI-RV	AAAGTCGACCGTCAGGCGCTGCCAGTGGTG
speF-SacI-FW	AAAGAGCTCGAAGAATCAGTTTCTCATGCG
speF-HindIII-RV	GGGAAGCTTCGACACCCATTTTATTGGATT
potA-FW	GCTTTCTGACTATAAAGC
potB-RV	GGACTACCGCGGATGATG
potF-FW	TTGCGAAGCTTCTATAGC
potI-RV	TGTGATGAGGTTGATTGC
SL prgI-FW	GGAGGACGCTATGTGCAG
SL prgI-RV	CGTGGCAATCGCCGAACC

SL potA-BamHI-FW	AAAGGATCCTATTTCACTTTGACACGC
SL potB-SphI-RV	AAAGCATGCGCGTGTGGATTTGTTAGC
SL potF-BamHI-FW	AAAGGATCCGGGTTCATTTGTTAACGG
SL potI-SphI-RV	AAAGCATGCCTTCCCTGAAAATGATGC
ssrB-red-FW	TAATTTTCGCGAGGGCAGCAAAATGAAAGAATATAAGATCGTGTAGGCTGGAGCTGCTTC
ssrB-red-RV	ACCAATGCTTAATACCATCGGACGCCCCTGGTTAATACTCCATATGAATATCCTCCTTAG
ssrB-FW	TATCTTAATTTTCGCGAGGGCAGCAAAATG
ssrB-RV	TGACCAATGCTTAATACCATCGGACGCCCC
SL potB-SphI-RV	AAAGCATGCGCGTGTGGATTTGTTAGC
SL potF-BamHI-FW	AAAGGATCCGGGTTCATTTGTTAACGG
SL potI-SphI-RV	AAAGCATGCCTTCCCTGAAAATGATGC
qsifA-FW	AATACCACCACCGCATACCC
qsifA-RV	TTACGAGGAACGCCTGAAAC
qsseB-FW	TGCCGAAGGGTATGGTGTT
qsseB-RV	TGACTTTTCCTGGGTATTTCTGG
qssaG-FW	AATTAGTGGATATGCTCTCCCACAT
qssaG-RV	GCTTTAATCATCGATTCTGGGTTG
qsseJ-FW	GGGGAAGTACATCGGCAAG
qsseJ-RV	CTGGTGAGAAGGGGTGTAAGATG
SL hilD-SacI-FW	CCCGAGCTCGAAAATGTAACCTTTGTA

SL hilD-EcoRI-RV	CCCGAATTCTGACAGATACAAAAAATG
SL prgH-red-FW	CTGCTGCTATCGAGAACGACAGACATCGCTAACAGTATATGTG TAGGCTGGAGCTGCTTC
SL prgH-red-RV	ACCAAGGTGTTGCCATAATGACTTCCTTATTTACGTTAAACAT ATGAATATCCTCCTTAG
SL prgH-F	TAATATGTGTTGAGACGC
SL prgH-R	TCCAGCGCCTCTGTTACC
SL prgH-EcoRI-FW	AAAGAATTCGAAACATCAAAAGAGAAG
SL prgH-HindIII-RV	CCCAAGCTTTTAAAGTGGGCTTGGGAA
Pro-invJ-XhoI-F	AAACTCGAGTTGGTTGCGCAAAGAAGG
invJ-BamHI-R	AAAGGATCCGGCGTCATCCTCCTCGCC
Pro-prgH-SalI	AAAGTCGACATCGTCAGTTTACCGCTC
SL prgH-BamHI-R	AAAGGATCCAAGTGGGCTTGGGAAATACC
SL prgI-BamHI-R	AAAGGATCCACGGAAGTTCTGAATAATG
prgH-Rev-BamHI	CCCGGATCCTGTCAGCAATGGAAACTC
Pro-ssaG-XhoI	AAACTCGAGCAAATGCTCAGGTAGGAGGGC
Rev-ssaG-BamHI	CCCGGATCCGATTTTAGCAATGATTCCACT
TNF-alpha-FW	CATCTTCTCAAAATTCGAGTGACAA

TNF-alpha-RV	TGGGAGTAGACAAGGTACAACCC
beta-actin-F	CCAGAGCAAGAGAGGTATCC
beta-actin-R	CTGTGGTGGTGAAGCTGTAG
mSlc7a1-F	AAAGCAGCCCTTCTCC
mSlc7a1-R	AGGCTCACTAGCCATCTGGA
mSlc7a2-F	TGATCCGGAGAAAAATTGTCA
mSlc7a2-R	CCCAAATTCAGCATAGCAAA
mNos2-F	CACCTTGGAGTTCACCCAGT
mNos2-R	ACCACTCGTACTTGGGATGC
mArg-1-F	AAGAAAAGGCCGATTCACCT
mArg-1-R	CACCTCCTCTGCTGTCTTCC
mArg-2-F	GGATCCAGAAGGTGATGGAA
mArg-2-R	AGAGCTGACAGCAACCCTGT
ODC-F	CAGCAGGCTTCTCTTGGAAC
ODC-R	CATGCATTTCAGGCAGGTTA
SRM-F	CGTTGGCTTCTCCAGCTCAAAG
SRM-R	AGGACTCCTTGAAGAGGCTCTC
SMOX-F	CAATGGCCTTTTGGAAGAGA
SMOX-R	TTACCATGCCGGAAGAACTC
