# Supplementary material for: Salmonella Typhimurium exploits host polyamines for assembly of the type 3 secretion machinery
Source: PLoS Biol. 2024 Aug 5;22(8):e3002731. doi: 10.1371/journal.pbio.3002731 (PMC11299824; doi:10.1371/journal.pbio.3002731)
Supplement: S1 Raw Images — (PDF) [file pbio.3002731.s019.pdf]

Corresponding to Figure 6D

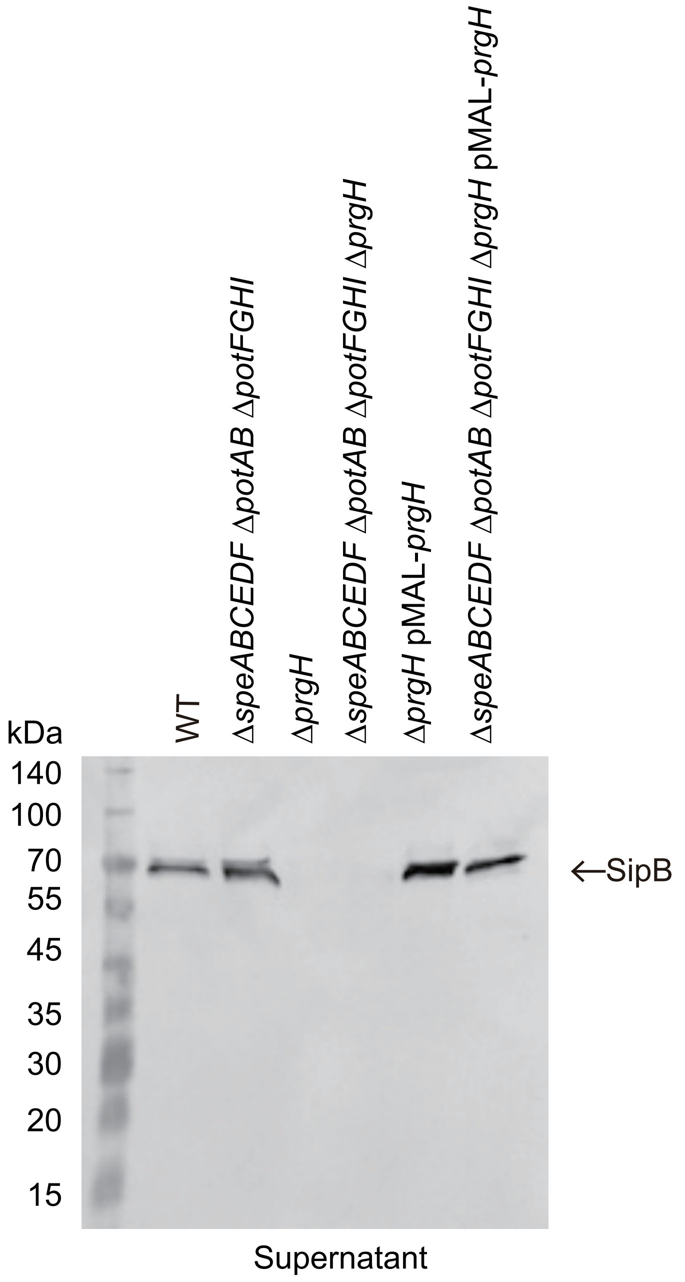

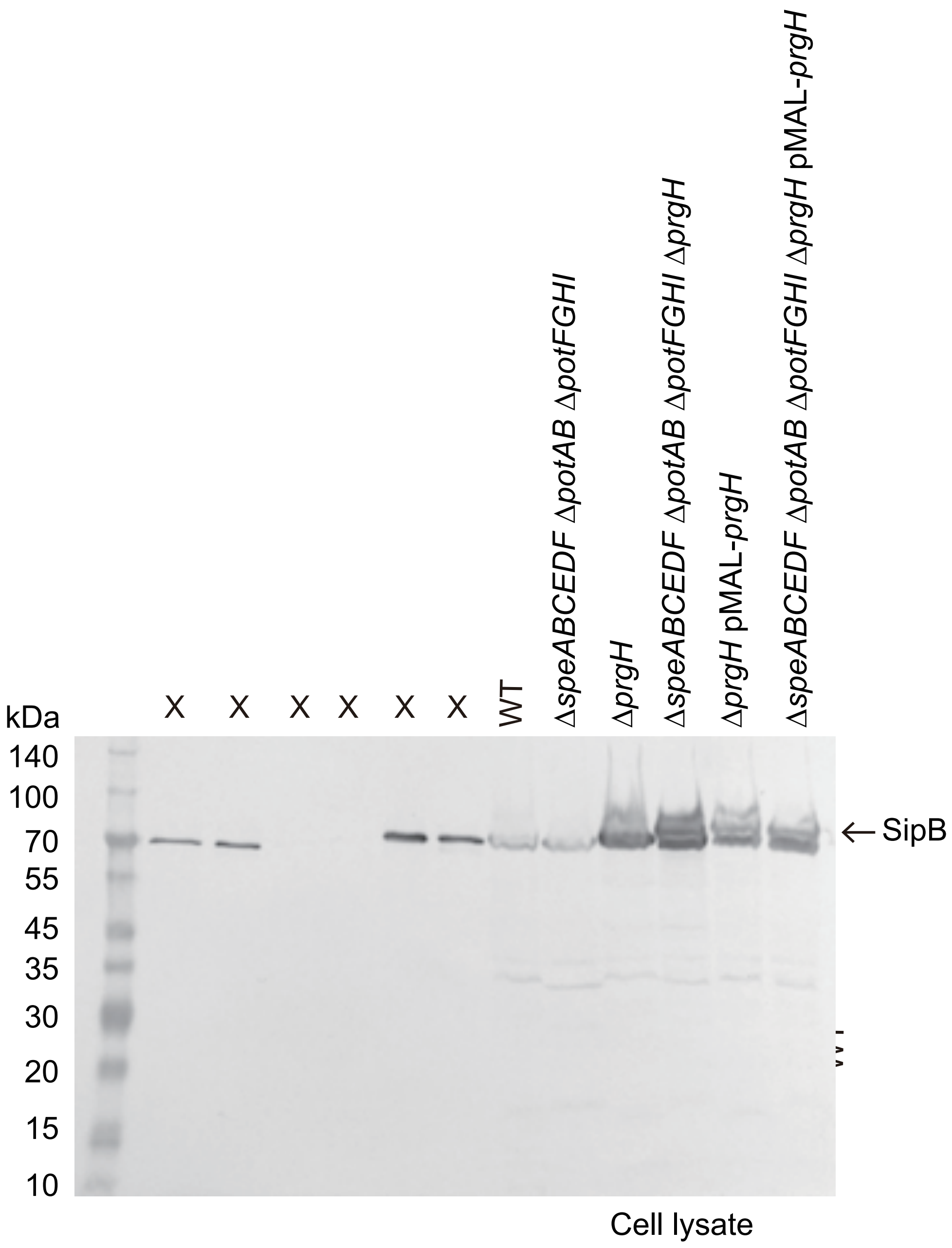

Corresponding to Figure 6E

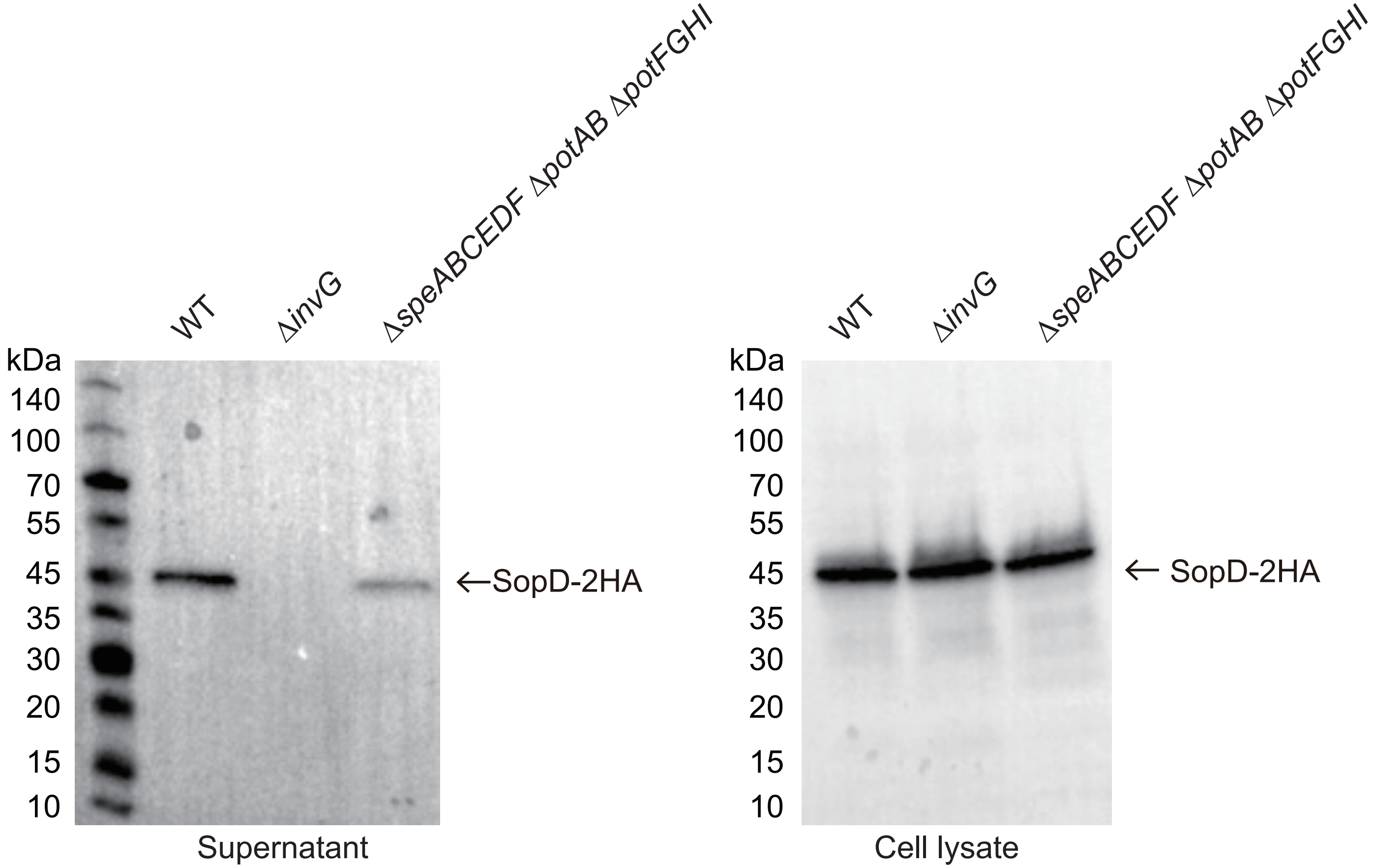

Corresponding to Figure 6F

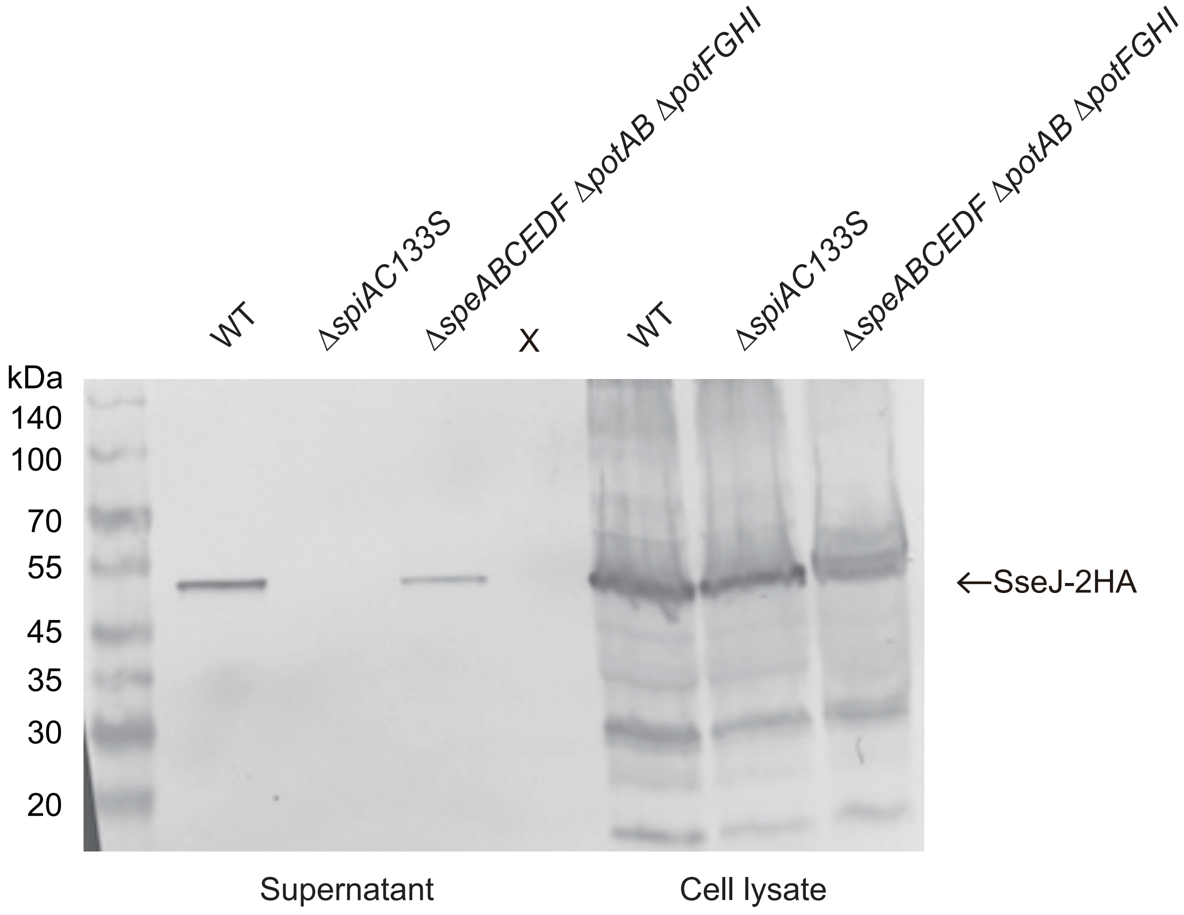

Corresponding to Figure 6G

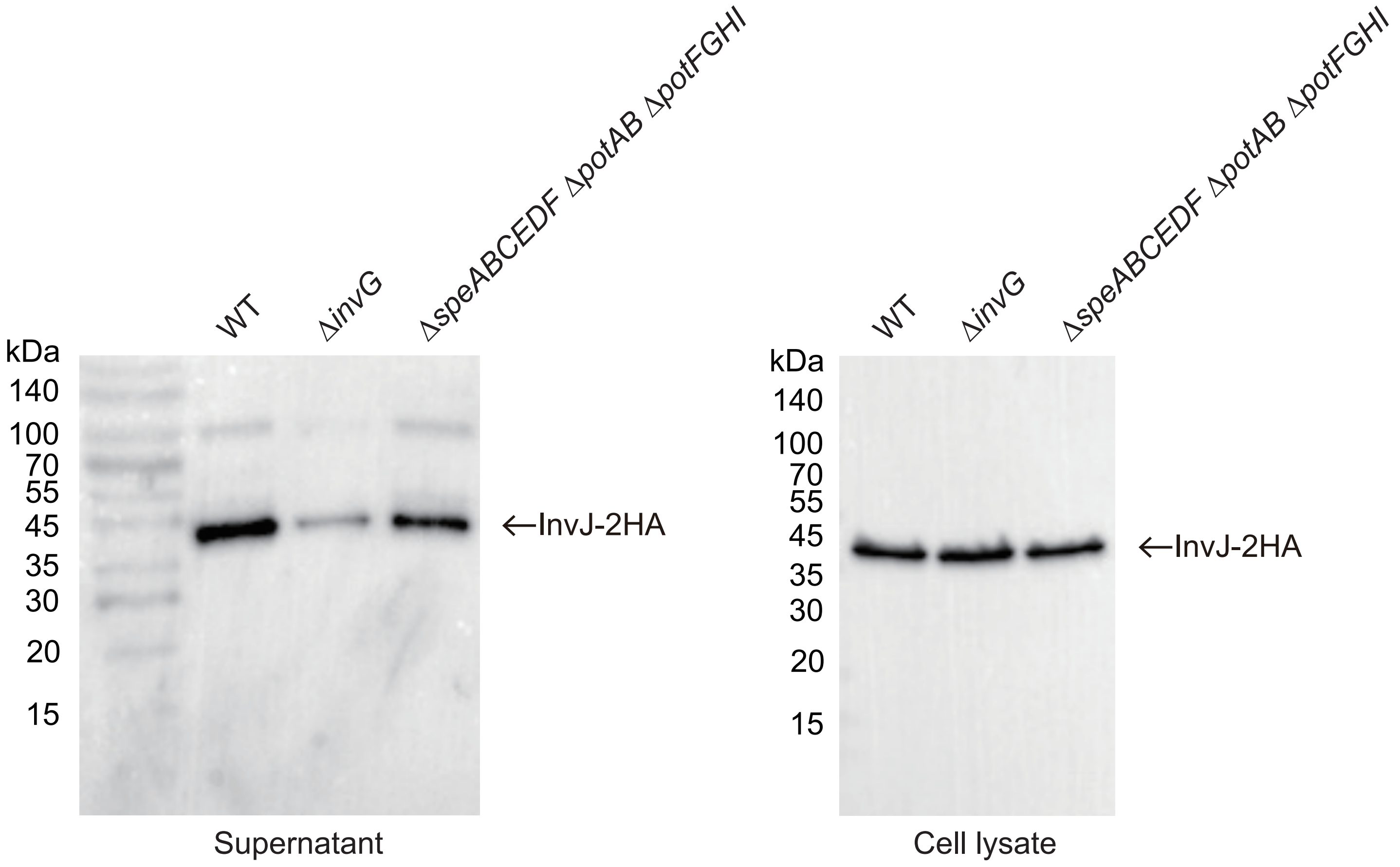

Corresponding to Figure S3C

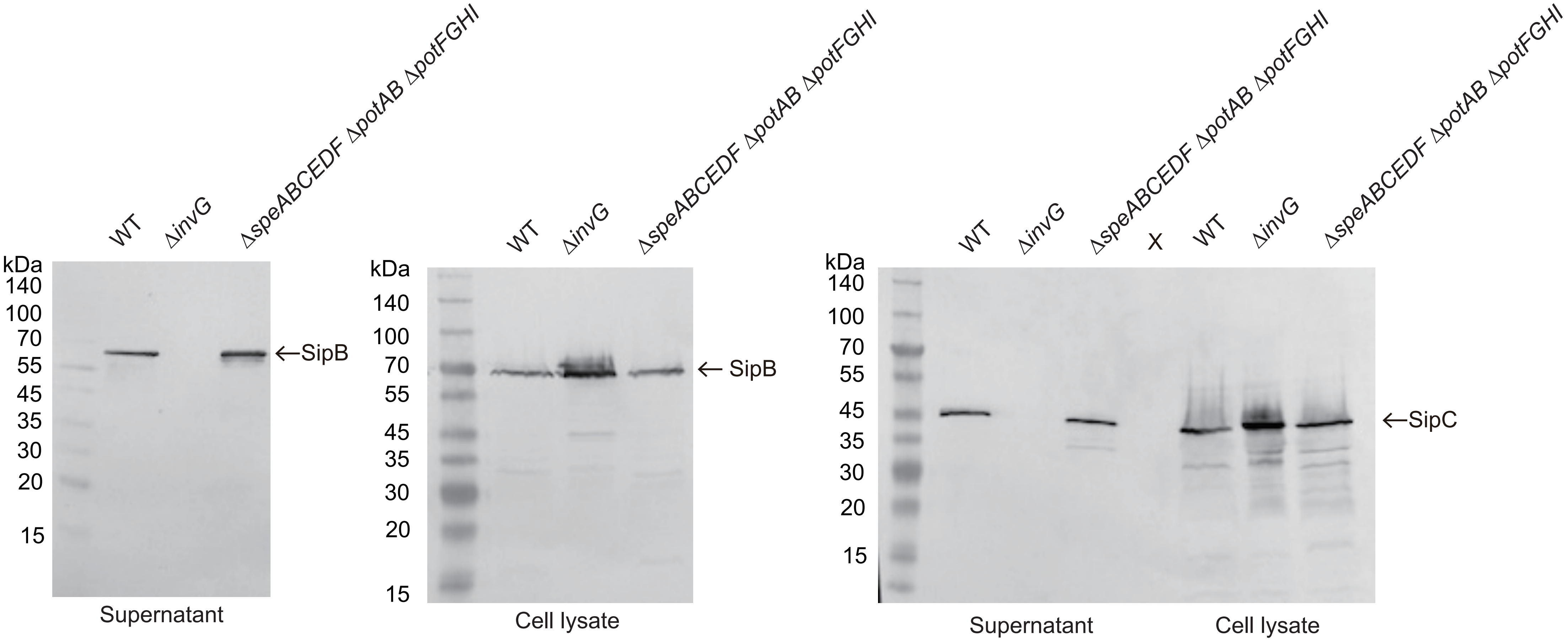

Corresponding to Figure S5C

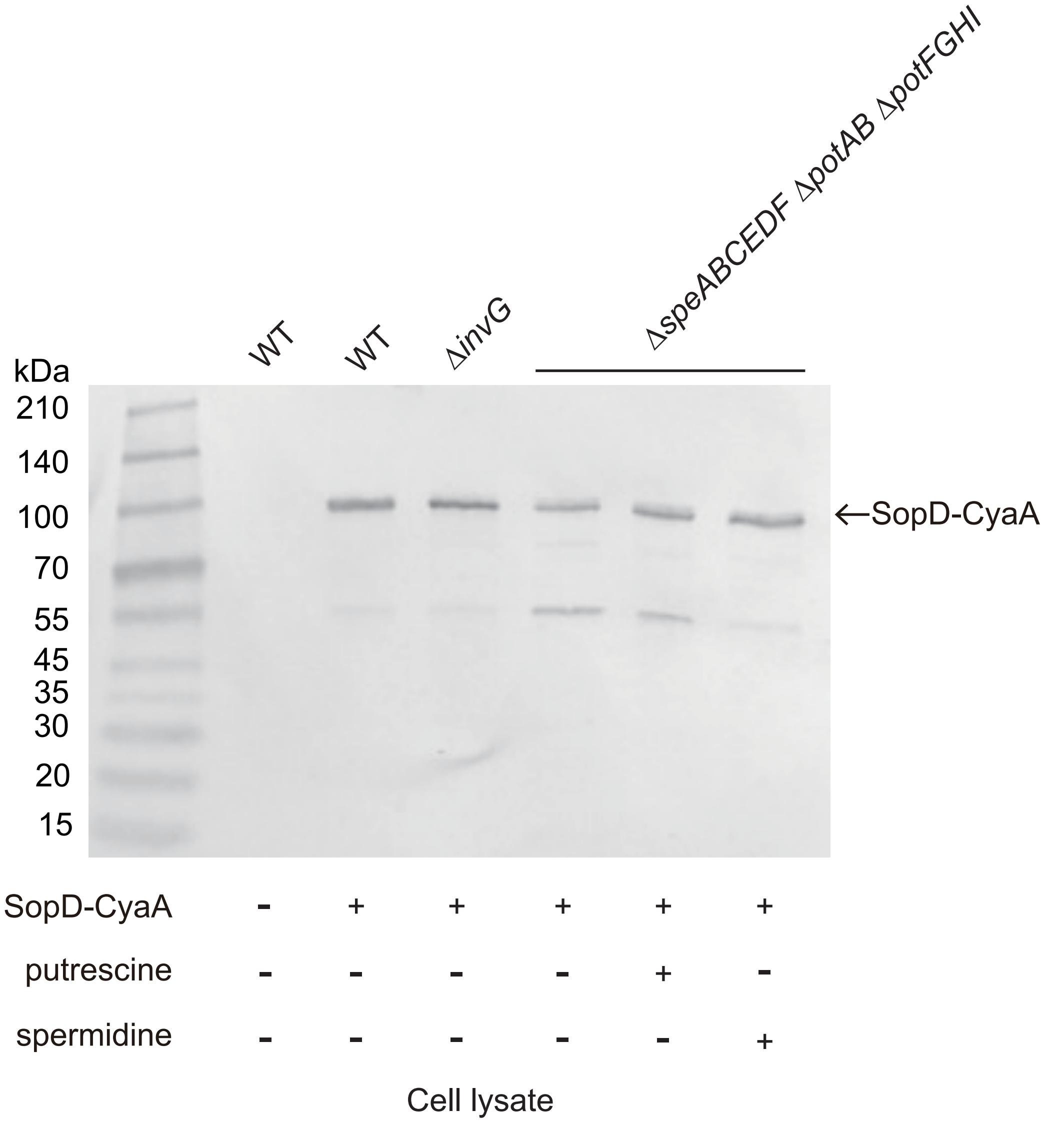

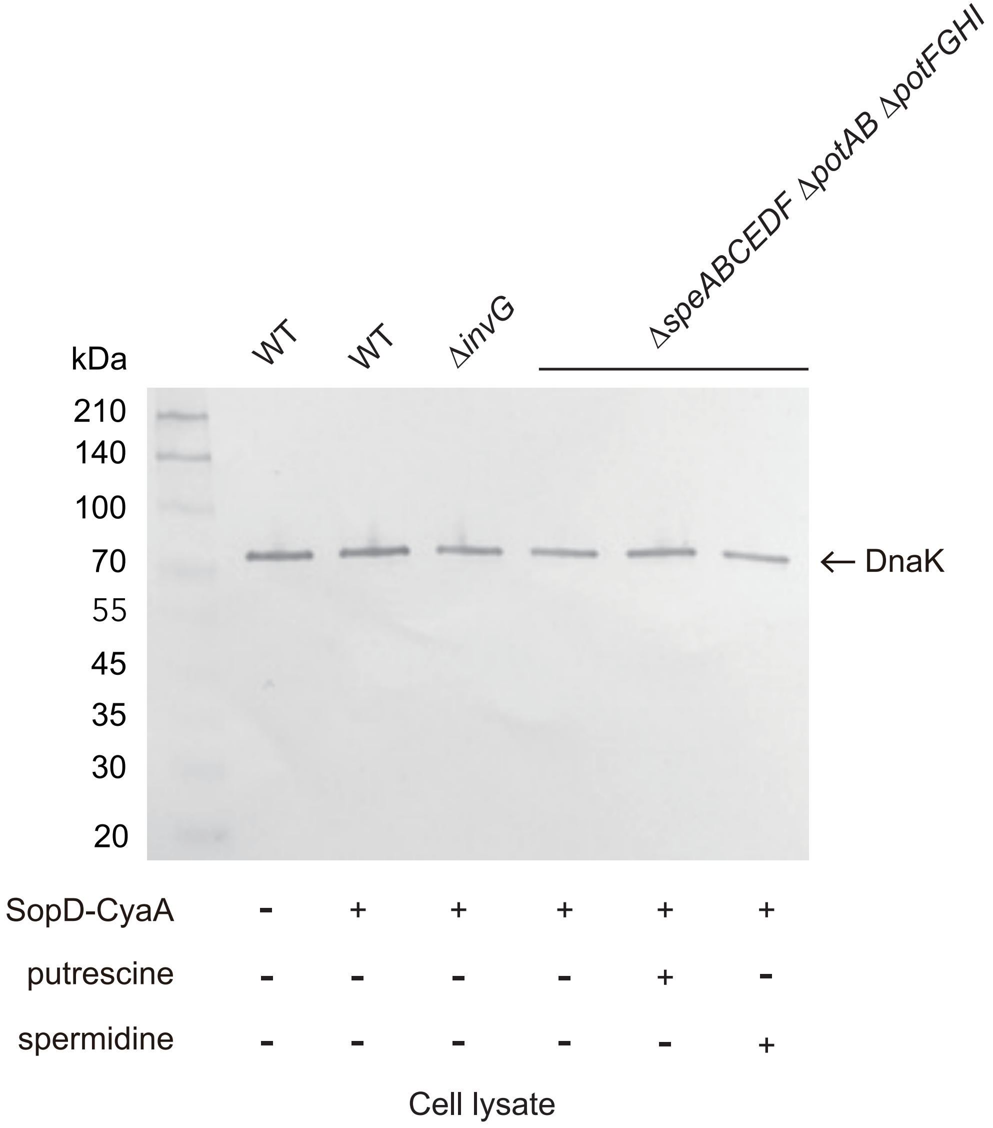

Corresponding to Figure S6B

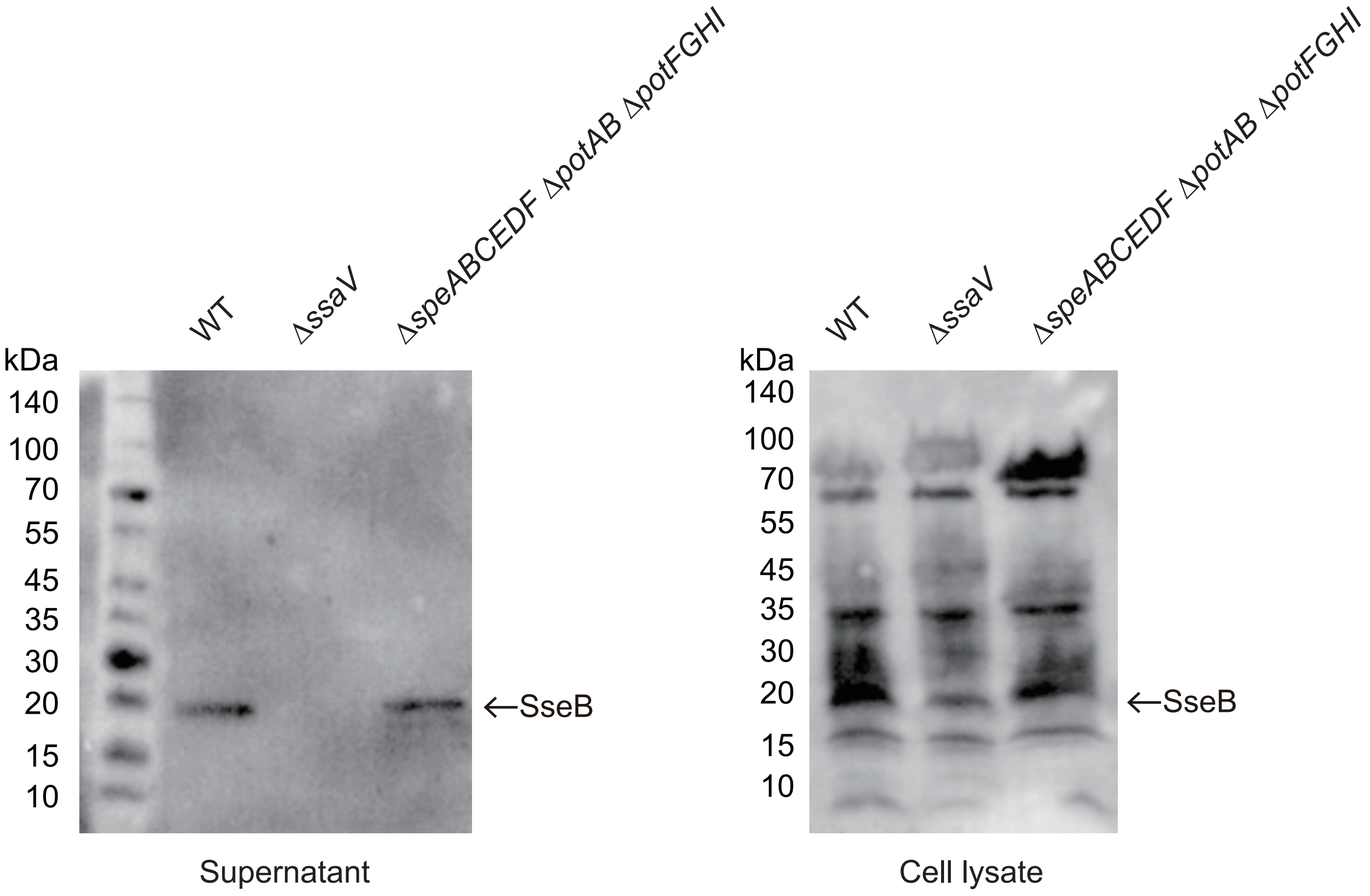

Corresponding to Figure S6I

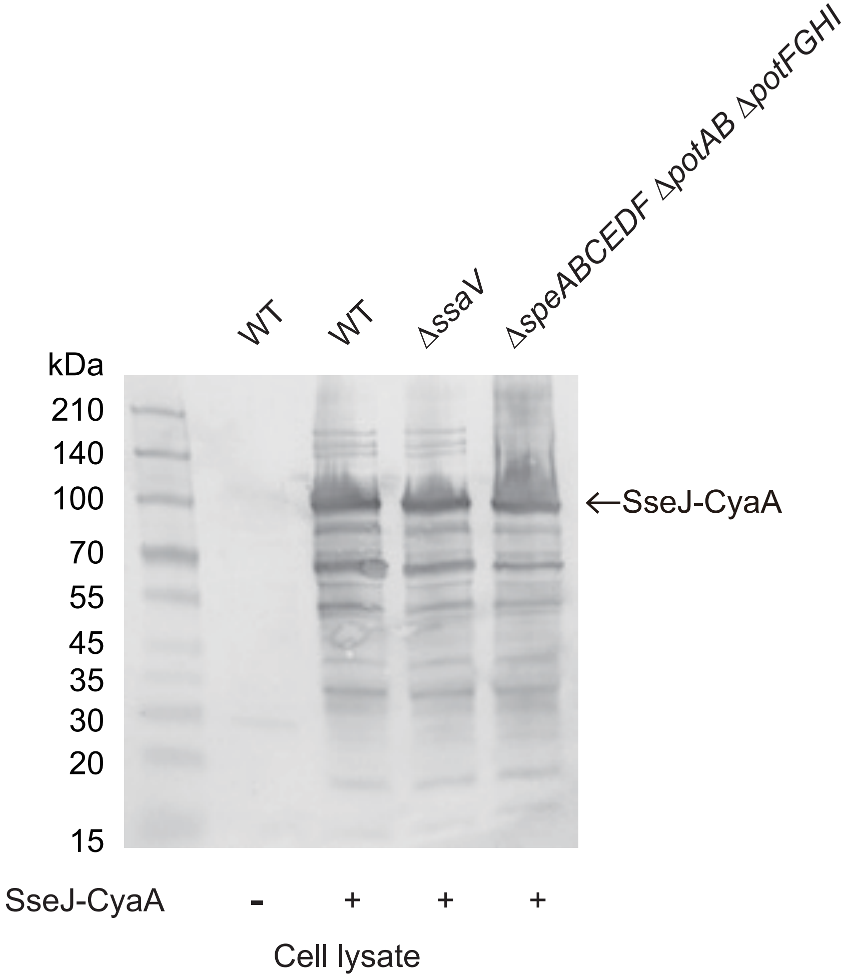

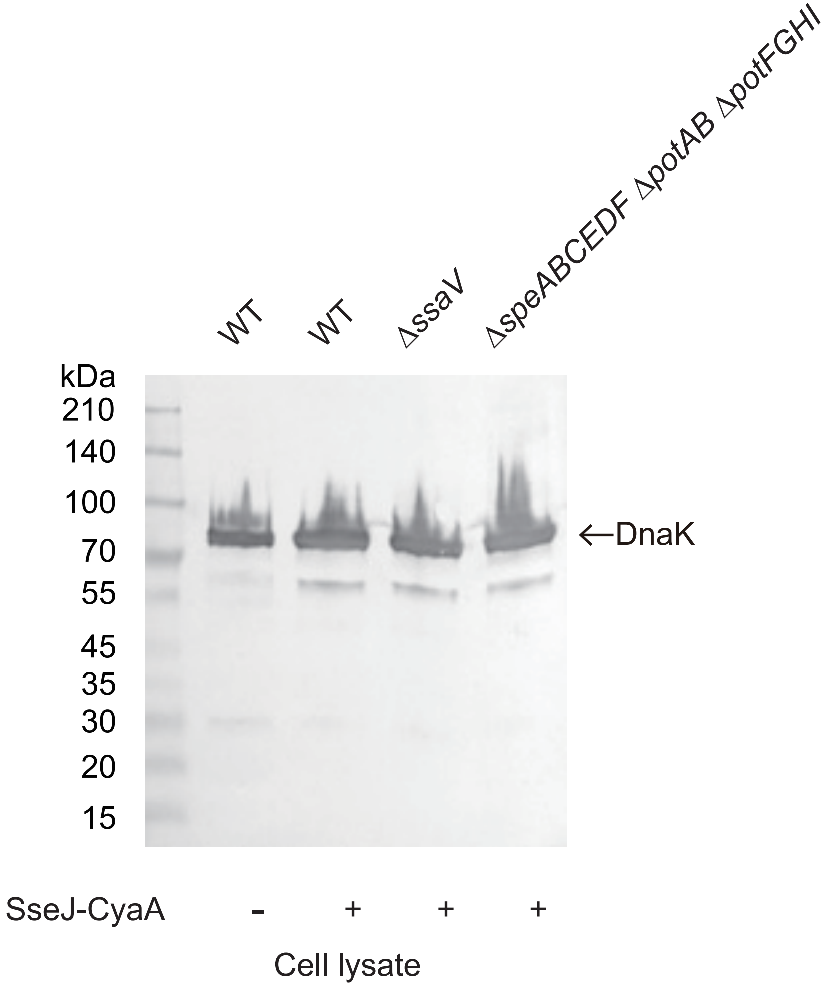

Corresponding to Figure S8C

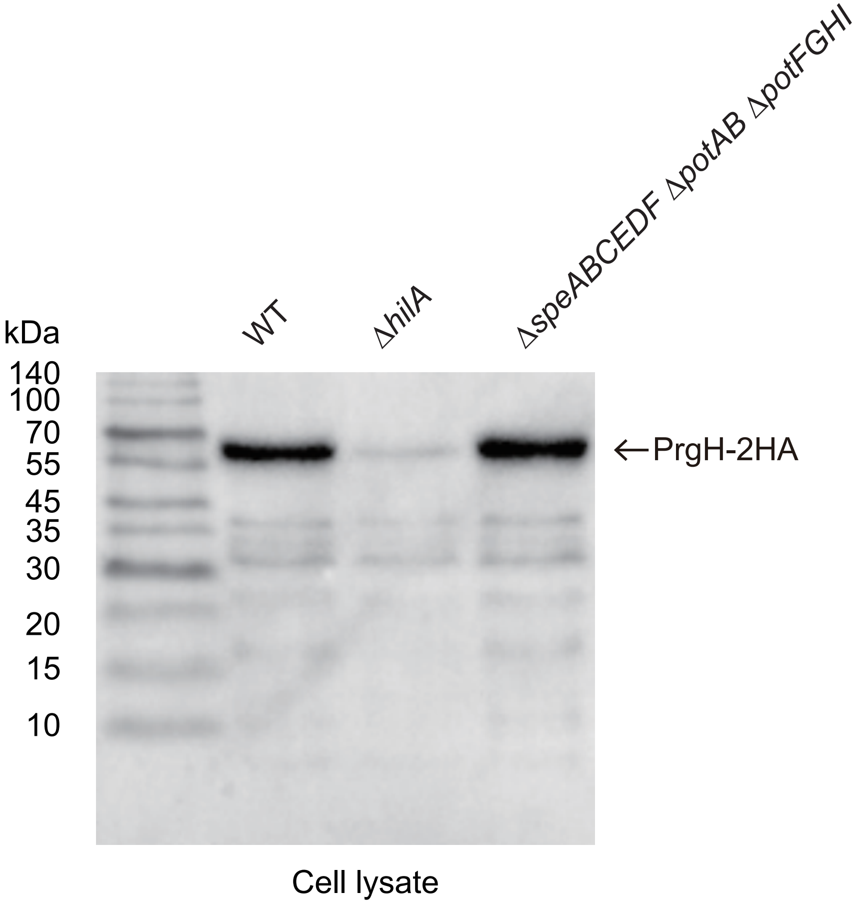

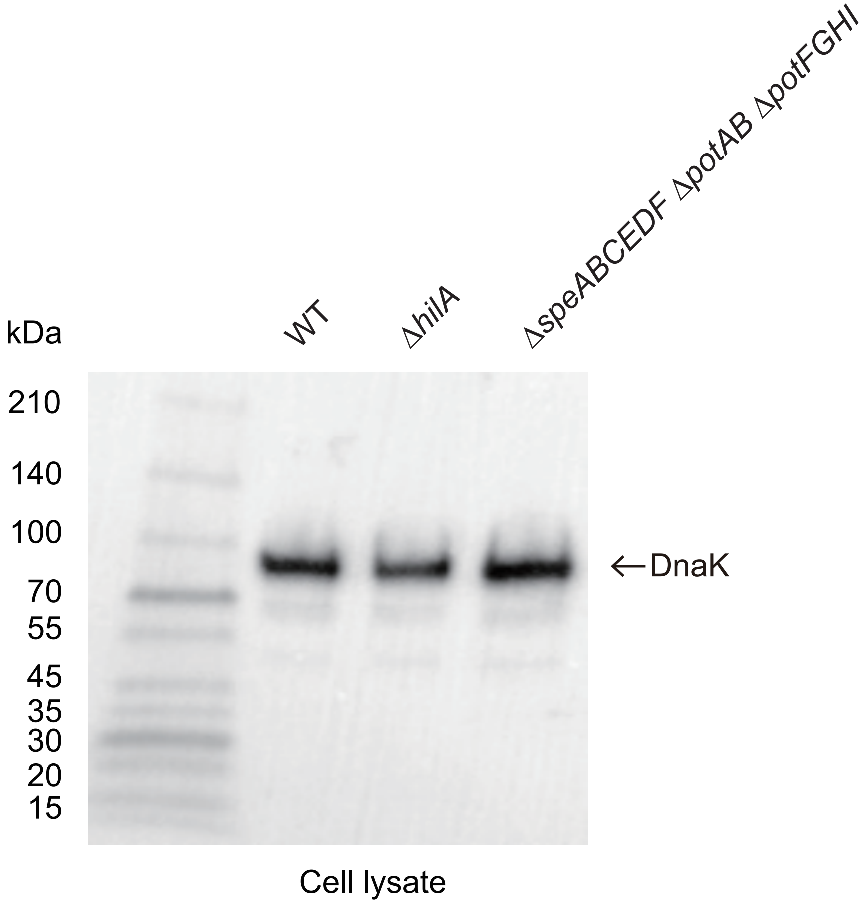

Corresponding to Figure S8D

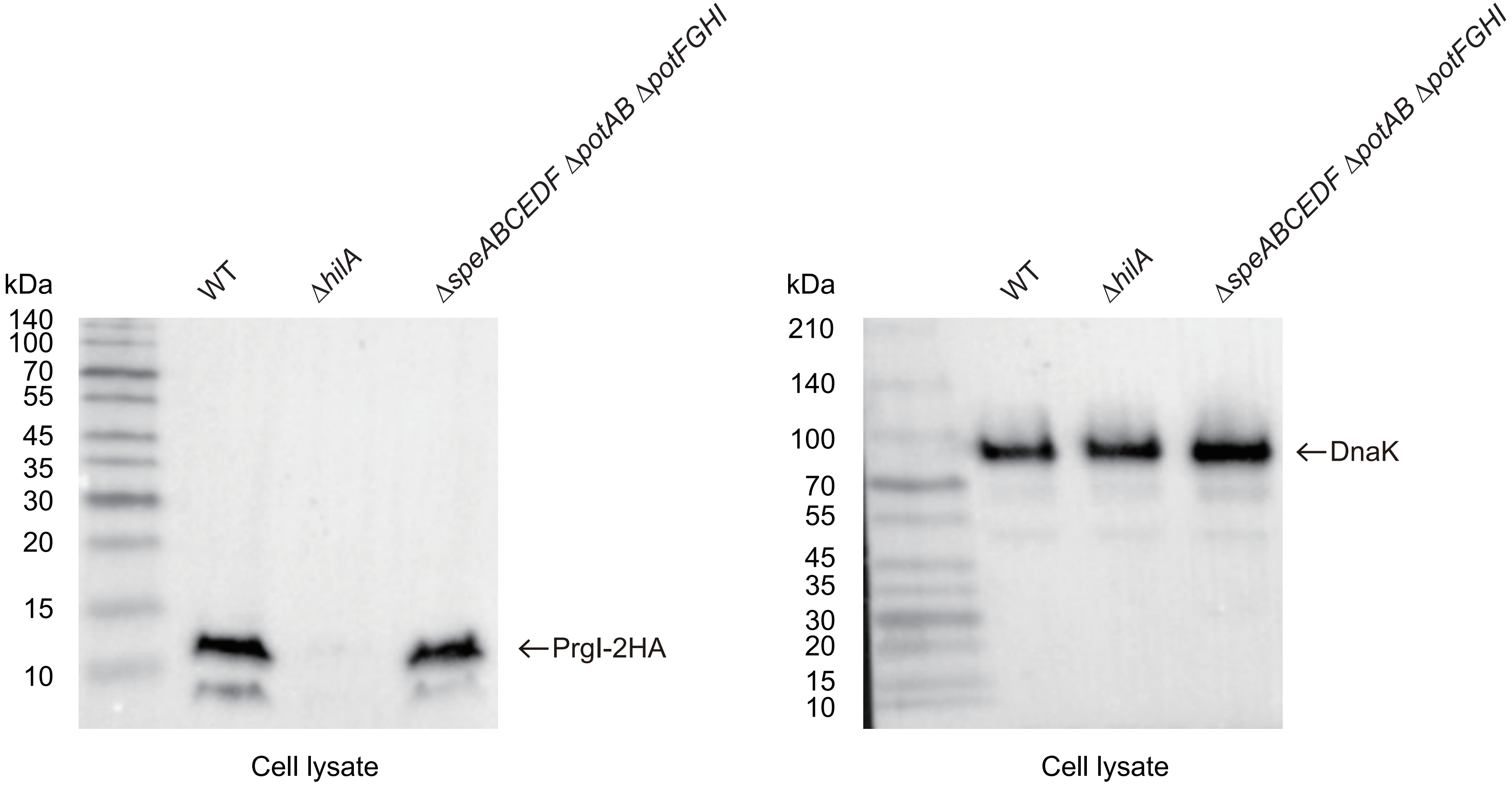

Corresponding to Figure S8E

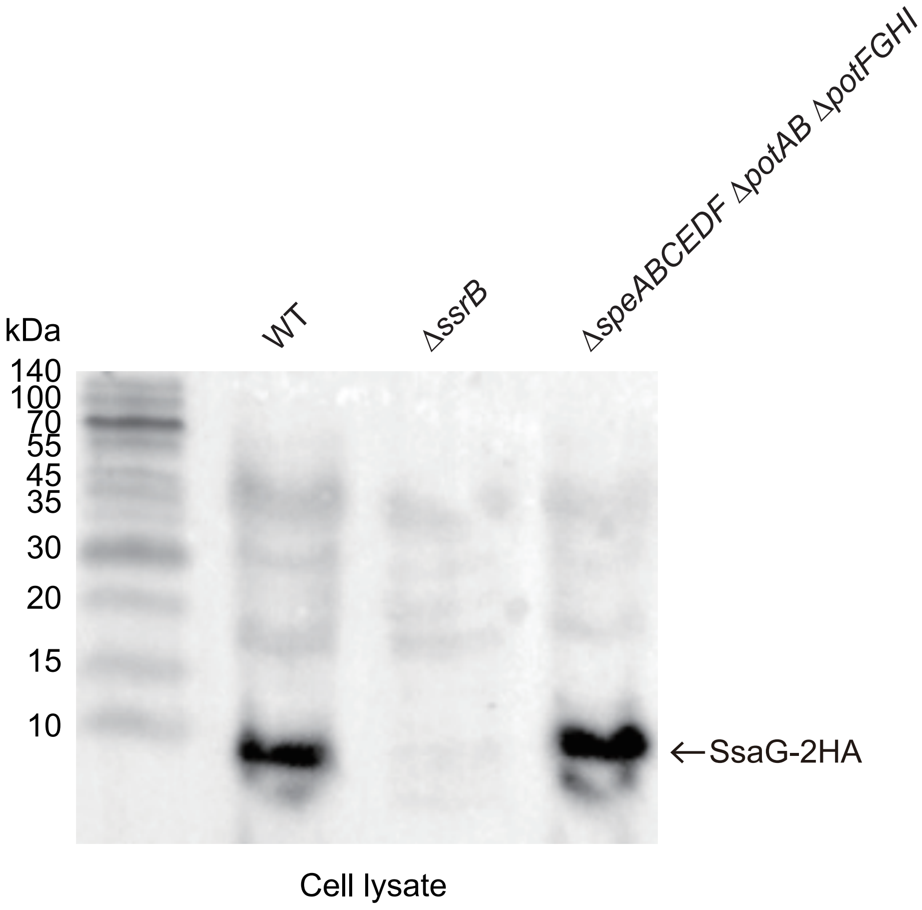

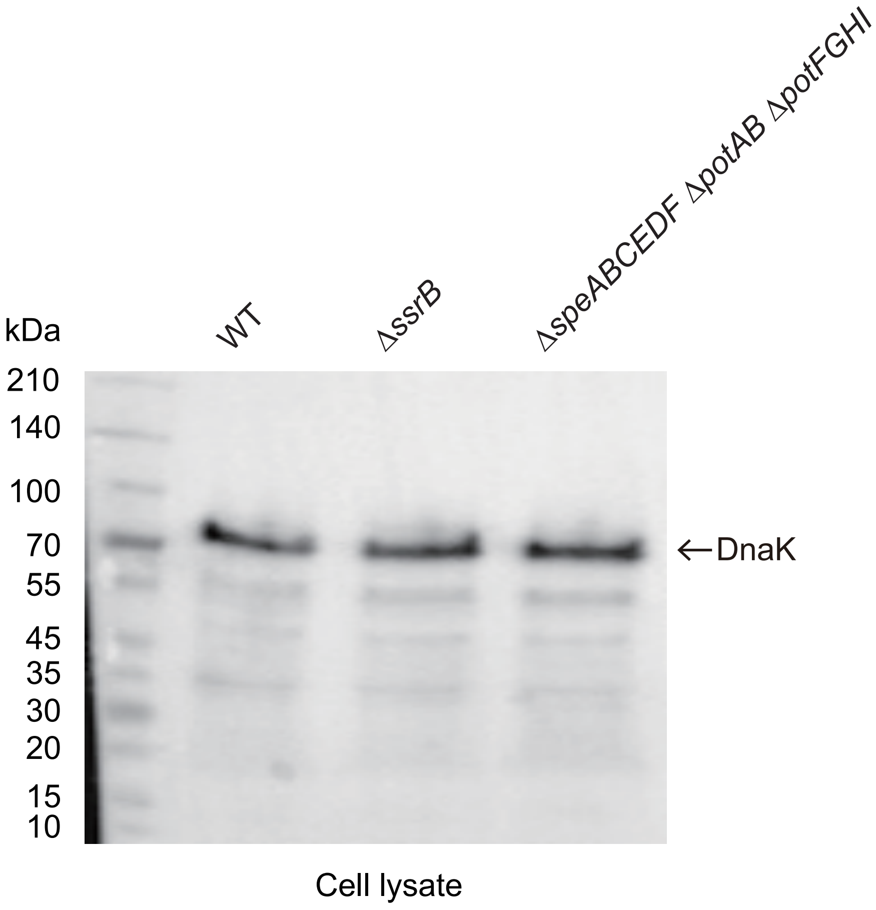

Corresponding to Figure S9A

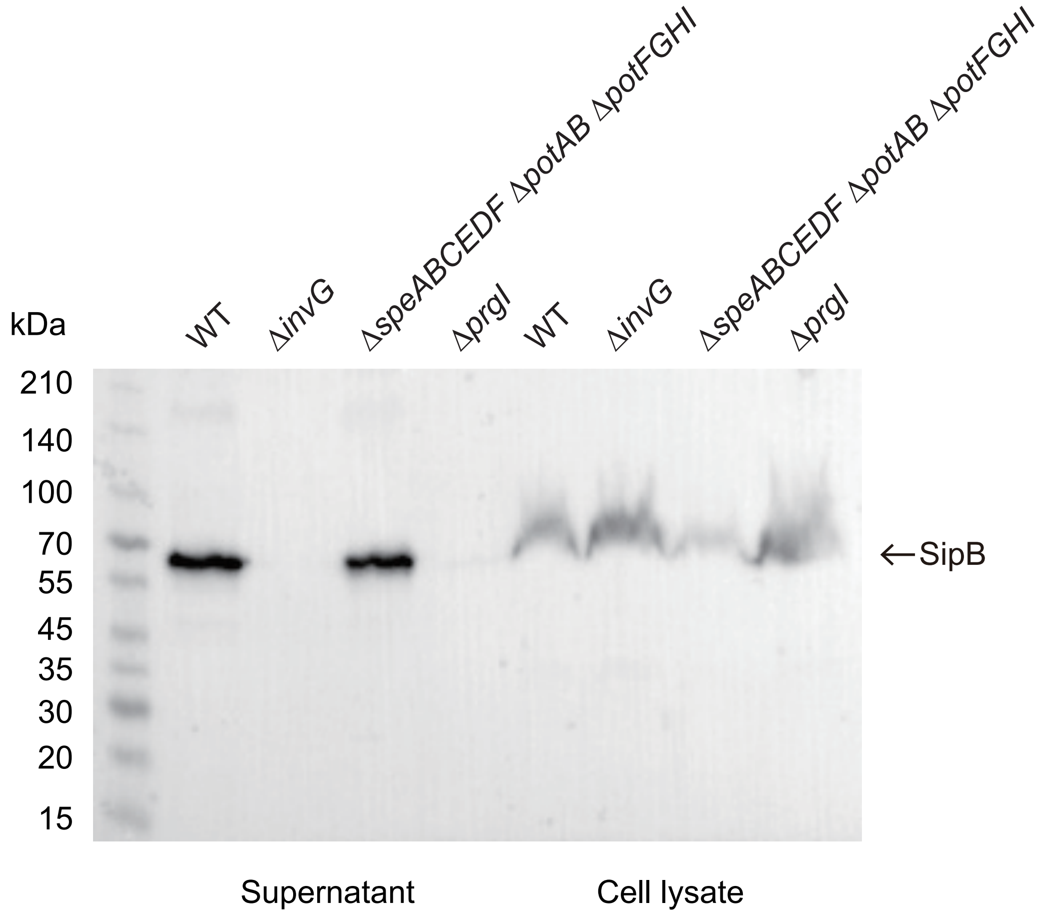

Corresponding to Figure S9B

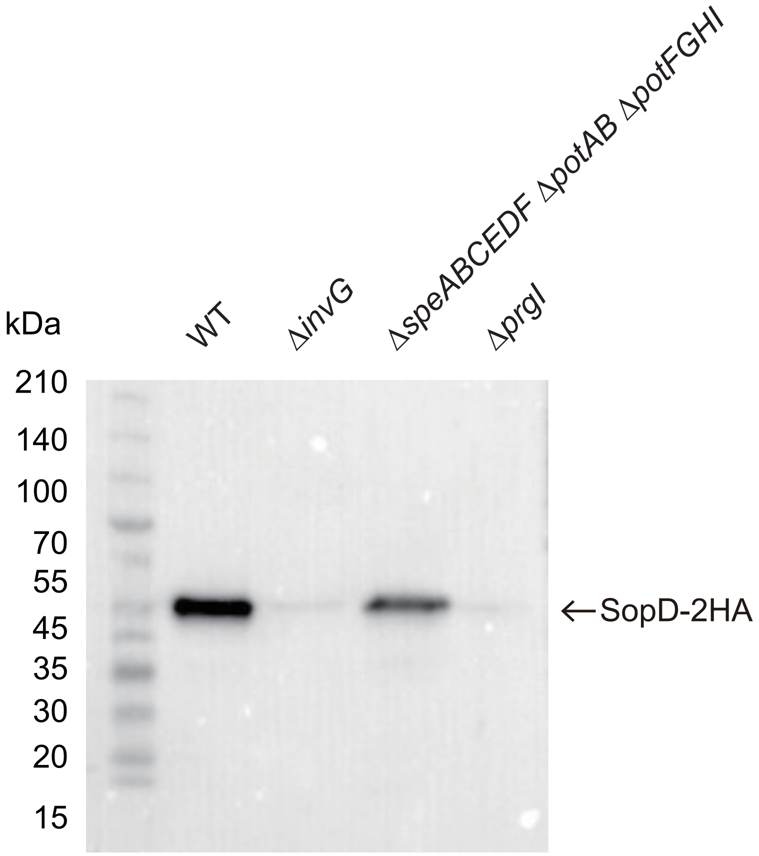

Supernatant

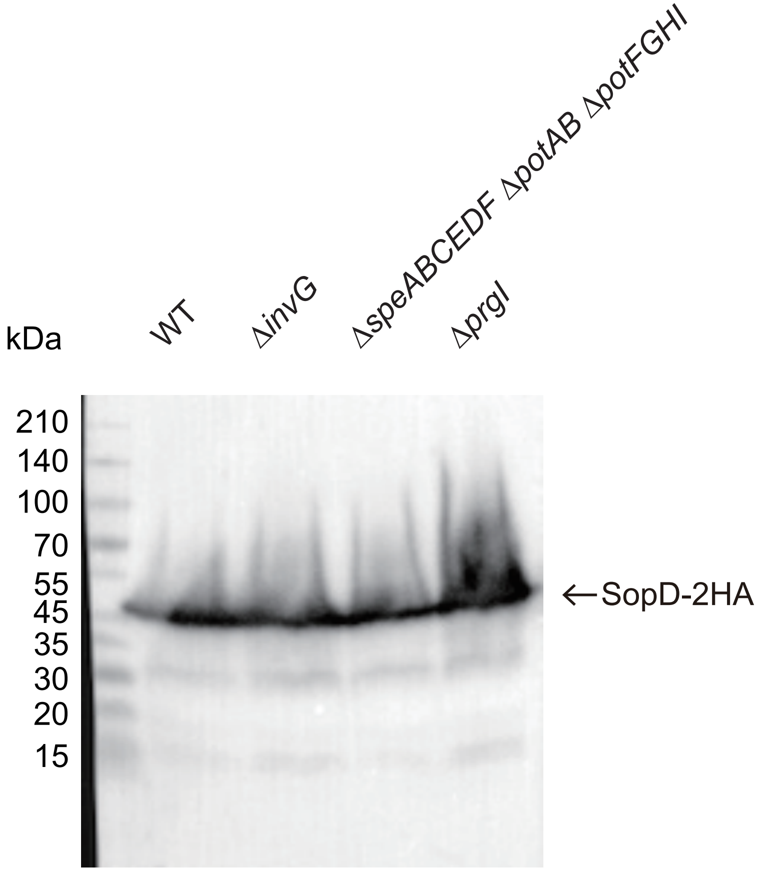

Cell lysate
