# Supplementary material for: Salmonella Typhimurium exploits host polyamines for assembly of the type 3 secretion machinery
Source: PLoS Biol. 2024 Aug 5;22(8):e3002731. doi: 10.1371/journal.pbio.3002731 (PMC11299824; doi:10.1371/journal.pbio.3002731)
Supplement: Attachment — Submitted filename: Response v1-8.pdf [file pbio.3002731.s020.pdf]

Point-by-point responses to Reviewers' comments
Manuscript ID#: PBIOLOGY-D-24-00105R1

Dear colleagues,

Thank you for the constructive feedback on our manuscript, PBIOLOGY-D-24-00105R1, entitled "*Salmonella* exploits host polyamines for assembly of the type 3 secretion machinery". In response to the input from the reviewers, we conducted several additional experiments. These mainly include i) measurement of intracellular polyamine levels, ii) mouse infection experiments to demonstrate the role of the polyamine homeostasis in T3SS-1 virulence, iii) invasion assays with the uptake mutant lacking both Pot transporters, iv) measurement of T3SS needle subunit expression, v) purification of the T3SS needle complex to further demonstrate the specific effects of host polyamines on the needle assembly of T3SS, and vi) measurement of polyamine contents in a DSS model mouse to clarify the link between host inflammation and polyamine levels. Moreover, we edited the manuscript text to make appropriate clarifications as the reviewers suggested. For convenience, changes in the revised manuscript are highlighted in red. Reviewer comments appear in bold, followed by our point-by-point responses.

Again, we would like to thank the reviewers for their valuable and constructive input, which we believe has improved the manuscript. We hope that the additions and adjustments described below satisfy their concerns.

We look forward to your response.

The Authors

Reviewer #1:
The manuscript "Salmonella exploits host polyamines for assembly of the type 3 secretion machinery" describes the effect of polyamines on Salmonella virulence and hints on how Salmonella induces host polyamine production to better survive at the infection sites. The manuscript follows a double path, one addressing the effect of polyamine on the assembly of the T3SS-1 (possibly translatable to T3SS-2) and the other one dealing with intracellular survival at later stages of infection, mediated by upregulation of polyamine production into the host and Salmonella SPD uptake.

Overall this is a promising study with interesting new results, however especially in the first part (Fig 1-4) the authors reasoning is not always obvious and the two paths described above often blend into each other. It would probably be helpful to rethink how to present the first part, also when it comes to the nomenclature of the different mutants and the weight the authors give to polyamine synthesis vs uptake contribution to Salmonella infection.

Response: We appreciate the overall positive comment by reviewer #1 and the time the reviewer spent to carefully consider this issue. In addition, we apologize if our presentation was sufficiently clear. As the reviewer suggested, to better present this work we have rearranged the first part of the original manuscript, splitting it into six distinct figures that show the results regarding the link between polyamine homeostasis and T3SS-dependent pathogenesis, the role of polyamine uptake in T3SS-1-, and T3SS-2-related virulence (Figures 3, 4, 5, S3, S5, and S6 in the revised manuscript).

Moreover, we apologize if our wording was insufficiently clear. We have carefully edited our manuscript to make it clearer. We hope that these modifications will satisfy the concerns of reviewer #1.

Specific comments:

1. The hypothesis that Salmonella could uptake polyamines through other transporters beside potABCD and potFGHI stems from the observation that PUT/SPD supplementation restores the growth defect in the ΔspeABCEDF ΔpotAB ΔpotFGHI mutant (Fig1). However there is no formal proof that this mutant can import polyamines and at what extent. It would be relatively easy to measure the bacterial intracellular polyamine concentration in this mutant grown with and without polyamine supplementation. This is particularly important since this mutant is then used as a proxy for polyamine uptake during in vivo infections. While this mutant partially recovers its colonization capacity when SPD is supplemented (Fig 2C-D), it is unclear at what extent this is due to SPD uptake, or to other reasons (e.g. stabilization of bacterial membrane; SPD effect on the host side).

Response: We thank the reviewer very much for suggesting this key experiment. We agree that our data from Figure 1 in the original manuscript are not sufficient to demonstrate the presence of other transporters or the uptake defects of the mutant. Thus, in response, we have measured the bacterial intracellular polyamine concentration of the

polyamine biosynthesis/transport-deficient mutant grown with and without polyamine supplementation. The results are presented in Figure R1 directly below this response. As evident from the results, the Δ*speABCEDF* Δ*potAB* Δ*potFGHI* can import exogenous polyamines through an as-yet-unidentified transporter. Therefore, we have included the following information: To explore the possibility, we investigated the intracellular contents of polyamines of *S*Tm strains grown in M9 supplemented with SPD or without supplementation. In M9 without SPD, intracellular SPD was detected in the WT but not Δ*speABCEDF* or Δ*speABCEDF* Δ*potAB* Δ*potFGHI* (Fig 1E). In contrast, intracellular SPD was detected at similar levels in all tested strains grown in M9 containing SPD. These results indicate that Δ*speABCEDF* Δ*potAB* Δ*potFGHI* can import exogenous polyamines through an as-yet-unidentified transporter. (Lines 119-125 of Page 5 in the revised manuscript)

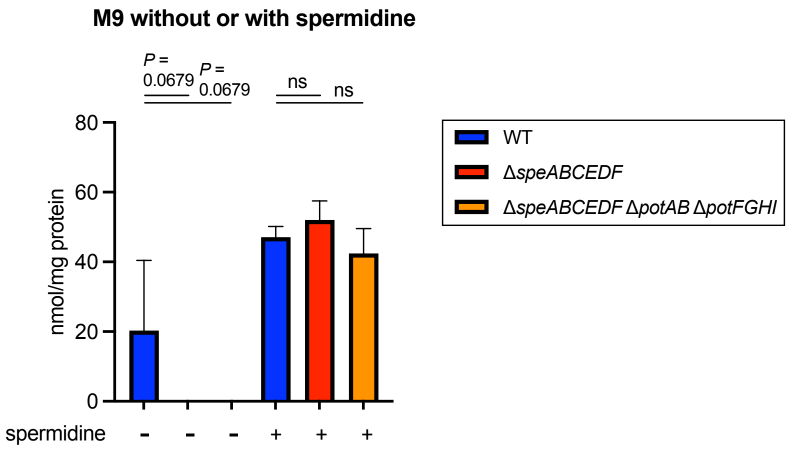

Figure R1. The Δ*speABCEDF* Δ*potAB* Δ*potFGHI* mutant can import exogenous spermidine through an as-yet-unidentified transporter. Measurement of intracellular spermidine contents from the WT, Δ*speABCEDF*, and Δ*speABCEDF* Δ*potAB* Δ*potFGHI* grown in M9 supplemented with spermidine at 3 mM. Values are means ± standard deviations of determinations in three independent experiments. ns, not significant; one-way ANOVA followed by Dunnett's multiple comparisons test.

2. All mouse infections were analysed at 4 days pi. In the Salmonella streptomycin mouse model this is a rather late stage, when multiple cycles of infection into the intestinal epithelium and reseeding into the intestinal lumen have happened. Moreover, it is known that bacterial mutants impaired in polyamine biosynthesis/transport are sensitive to multiple stressors (e.g. pH, oxidative stress).

With this in mind, it becomes unclear at which stage of infection polyamines might play a role in Salmonella colonization. An infection kinetic experiment which looks at earlier stages of infection would be helpful to tease apart how polyamine uptake would affect the infection progression. For example, does the ΔspeABCEDF ΔpotAB ΔpotFGHI mutant colonize the lumen at similar levels as the wt at earlier time points (for example 12 hours p.i.)? If this is the case, would the mutant be impaired at infecting the cecum tissue (as suggested by the defective T3SS-1 assembly)?

Response: We appreciate this excellent suggestion and agree with the reviewer that the polyamine biosynthesis/transport-deficient mutant is sensitive to numerous stressors. Therefore, to demonstrate the link between polyamine uptake and T3SS-1-dependent gut infection, we investigated the in vivo virulence of the ΔspeABCEDF ΔpotAB ΔpotFGHI mutant on day 2 after infection, in which the induction of gut inflammation depends on the T3SS-1 activity (Barthel et al., Infect Immun 2003). The results are presented in Figure R2 below this response. As evident from these data, the ΔspeABCEDF ΔpotAB ΔpotFGHI mutant is impaired in T3SS-1 compared to the WT. Furthermore, the defect is reversed by supplementation with spermidine, indicating that uptake-dependent polyamine homeostasis has a critical role in T3SS-1–dependent pathogenesis in vivo. The new data presented in Figure 3E of the revised manuscript further demonstrate the link between polyamine homeostasis and the functional expression of T3SS-1.

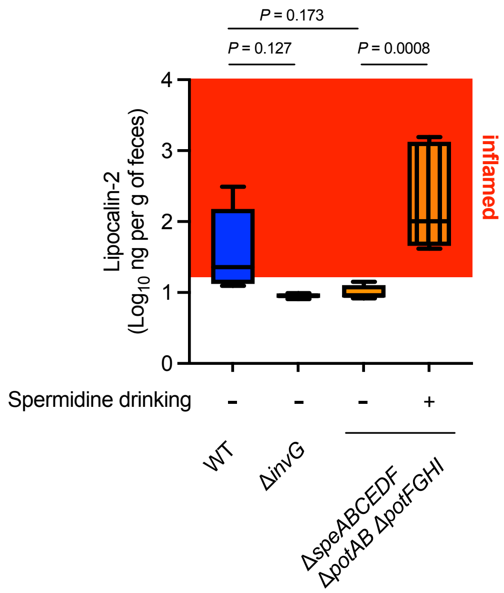

Figure R2. Uptake-dependent polyamine homeostasis contributes to T3SS-1–dependent gut inflammation in a mouse model. Streptomycin-pretreated C57BL/6

mice were infected by gavage with the WT, Δ*invG*, or Δ*speABCEDF* Δ*potAB* Δ*potFGHI*, or Δ*speABCEDF* Δ*potAB* Δ*potFGHI* with spermidine administered in drinking water for 2 days (n = 5, respectively). Lipocalin-2 levels in feces were determined. The box plot with whiskers shows the range from minimum to maximum values, and the black bars indicate medians. One-way ANOVA followed by Dunnett's multiple comparisons test.

3. How would polyamines facilitate the assembly of the T3SS-1? It would be interesting to at least discuss a possible mechanism. Moreover, considering that in a normal infection setting Salmonella arrives at the infection site with its own set of intracellularly synthesized polyamines, does the Salmonella transporter mutant ΔpotAB ΔpotFGHI also show signs of impaired T3SS-1 assembly? This mutant seems to have no colonization defect in the cecal content, but rather a defect in spreading to systemic sites. Would this suggest that polyamine uptake might have a more prominent role in bacterial intracellular survival and/or spread?

Response: We thank the reviewer for the helpful suggestion. We agree that a discussion of the possible mechanism by which polyamines facilitate the assembly of the T3SS-1 needle would be very interesting. Therefore, we have included the following sentences in the Discussion section in the revised manuscript: How may polyamines facilitate needle assembly? Polyamines are ubiquitous in nature and mammalian cells at high levels (greater than millimolar concentrations), and it is notable that polyamines can interact with proteins by serving as counterions to anionic targets. Indeed, SPD and SPM play regulatory roles in microtubule assembly in cells by controlling the transition of microtubule structures between straight and curved, leading to the formation of bundles [48, 49]. Moreover, it is shown that polyamines control the assembly of neuronal receptors while also acting a gatekeeper [50-53]. Based on the previous observations, it is reasonable to speculate that SPD may interact with the needle subunits through ionic binding, thereby facilitating the assembly. Moreover, the low isoelectronic point values of the needle subunits, PrgI and SsaG, might facilitate the interaction with polyamines that possess a cationic nature. Furthermore, one may consider an alternative scenario: "Indirect involvement". The Δ*speABCEDF* Δ*potAB* Δ*potFGHI* has pleiotropic effects [23, 25, 29]. Thus, in this mutant, the needle is assembled, but may fall out of the basal body during bacterial growth. Namely, spermidine might interact with as-yet-unidentified proteins, leading to the needle assembly. (Lines 698-712 of Pages 26-27 in the revised manuscript)

In addition, the reviewer's comment regarding the transporter mutant Δ*potAB*

Δ*potFGHI* is very important for clarifying the role of the Pot transporter in *Salmonella* pathogenesis. We thus investigated the invasiveness of the transporter mutant Δ*potAB* Δ*potFGHI*. As shown in Figure R3 below, the invasiveness of Δ*potAB* Δ*potFGHI* into HeLa cells was impaired. Furthermore, the defect was reversed by introducing a plasmid expressing PotAB, whereas SPD supplementation did not lead to reversal, suggesting that the PotABCD transporter contributes to invasiveness in a polyamine uptake-independent manner. In addition, to determine the T3SS-1 activity in vivo in the Δ*potAB* Δ*potFGHI*, we investigated it ability to induce T3SS-1-dependent gut inflammation in a mouse model. As shown in Figure R4 below, the Δ*potAB* Δ*potFGHI* exhibits similar activity in inducing gut inflammation to the WT strain. As a result of this excellent suggestion by the reviewer, we noticed that the PotABCD transporter has dual roles in cell invasion: one dependent on polyamine uptake and the other independent of it. Moreover, based on the results of mouse infection experiments, it is notable that uptake-dependent polyamine homeostasis plays a critical role in T3SS-1 activity in vivo.

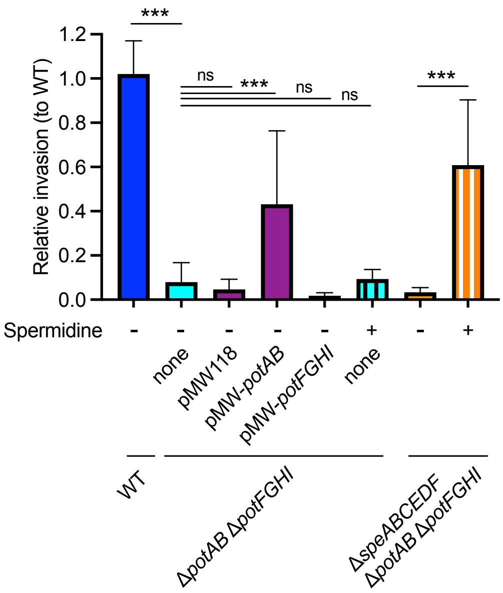

Figure R3. PotABCD transporter contributes to cell invasion in a polyamine uptake-independent manner. HeLa cells were infected with the indicated *S*Tm strains, and the number of invading bacteria was determined by a gentamicin protection assay. The invasion levels relative to the WT strain are shown from at least three independent experiments. Bars represent means ± SD. ns, not significant; $^{***}P < 0.001$; one-way ANOVA followed by Dunnett's multiple comparisons test.

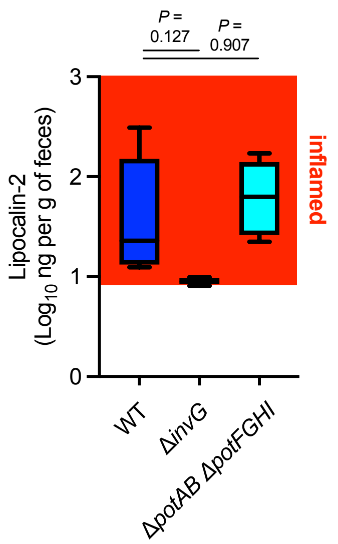

Figure R4. Uptake-dependent polyamine homeostasis contributes to T3SS-1–dependent gut inflammation in a mouse model. Streptomycin-pretreated C57BL/6 mice were infected by gavage with the WT or Δ*invG* or Δ*speABCEDF* Δ*potAB* Δ*potFGHI* or Δ*speABCEDF* Δ*potAB* Δ*potFGHI* with spermidine drinking for 2 days (n = 5, respectively). Lipocalin-2 levels in feces were determined. The box plot with whiskers shows the range from minimum to maximum values, and the black bars indicate medians. One-way ANOVA followed by Dunnett's multiple comparisons test.

4. The results with DFMO supplementation are very interesting and potentially impactful for therapeutic treatments, considering this is an already approved drug. This aspect could probably be stressed out more in the abstract as a point of novelty. Also, is the polyamine content reduced in macrophages in the presence of DFMO? How would DFMO affect the macrophage differentiation status? Please note that there is a recent pre-print showing similar/overlapping results (https://www.biorxiv.org/content/10.1101/2023.09.29.560257v2)

Response: We thank the reviewer for pointing this out. As the reviewer requested, we modified the abstract in the revised manuscript as follows: The decline in polyamine levels caused by difluoromethylornithine, which inhibits host polyamine production, attenuated *S*Tm colonization, (Lines 36-38 of Page 2 in the revised manuscript)

We also confirmed the effect of DFMO in macrophages, in which polyamine levels are drastically decreased compared to control, untreated macrophages. We included the results in Supplemental Figure 6 of the revised manuscript, as follows: DFMO

treatment drastically reduced the levels of PUT and SPD, but not SPM, in RAW264.7 cells (S9 Fig). (Lines 550-551 of Page 21 in the revised manuscript)

Earlier work demonstrated that a deficiency of macrophage-derived ornithine decarboxylase (ODC) has an inhibitory effect on M1 macrophage activation (Hardbower et al., Proc Natl Acad Sci USA 2017; PMID: 28096401). Therefore, DFMO, a potent inhibitor of ODC, leads to M1 activation along with enhanced production of nitric oxide, thereby suppressing *Salmonella* infection. Thus, we included the following sentence in the revised manuscript: Indeed, earlier work demonstrated that the deficiency of macrophage-derived ODC enhances M1 macrophage activation [71], indicating that DFMO leads to M1 activation along with enhanced production of nitric oxide and thereby suppresses *S*Tm infection. (Lines 781-784 of Pages 29-30 in the revised manuscript)

5. The authors discuss the possibility that Salmonella induces macrophage differentiation towards an M2 status through upregulation of polyamines, which would facilitate Salmonella intracellular replication. In principle the authors have all necessary tools available to complement their studies by also testing this hypothesis, for example following Salmonella infection in primary macrophages.

Response: We appreciate this excellent suggestion. Unfortunately, however, we presently have no way to perform the experiments with primary macrophages in our laboratory. We acknowledge that this direction would help us examine our hypothesis. Therefore, this is an important topic for future work. Again, we would like to thank the reviewer for the very valuable suggestion.

Reviewer #2:

Polyamines are ubiquitous molecules with diverse, but poorly understood functions. Polyamines have been shown to both influence and be a target of bacterial infections. In this study, Miki and colleagues describe the role of bacterial polyamine levels in infection by Salmonella enterica, an influence on the two Salmonella type III secretion systems (T3SS), and an effect of Salmonella infection on host polyamine biosynthesis. Together, the results indicate that sufficient polyamine levels are required for infection and that Salmonella actively promote increased levels.

This is a nice manuscript at the interface of bacterial virulence and host response. The results are presented in a clean and logical manner. The striking effect of polyamine levels on infection and the manipulation of host polyamine synthesis (Fig.

1, 2, 3, 5, 6 and corresponding Suppl. Figures) are worked out clearly; however, similar results were already shown in earlier publications, including for Salmonella. The completely novel observation the specific effect on T3SS needle formation is less obviously convincing, see comments below.

Response: We would like to thank reviewer #2 for the positive comment and for taking the time to carefully consider this issue. Please see our responses to the reviewer's comments below.

Major comments:

1. There are several publications that have described links between host polyamines and bacterial infections, including for Salmonella. This previous knowledge is partially discussed in the discussion part, but should already be presented more clearly in the introduction. This includes, but is not limited to:

- **Salmonella infection increases Arg-1 and Arg-2 expression (refs. 49, 50, mentioned in lines 575-577)**
- **Spermidine increases T3SS expression in Pseudomonas aeruginosa (DOIs 10.1128/spectrum.00644-22, 10.1371/journal.pone.0001291)**
- **Polyamine levels strongly influence protein expression in Salmonella, including the T3SS components (ref. 23, mentioned in line 532)**
- **A very recent paper by Nair et al. (DOI 10.1016/j.micres.2024.127605), which shows multilayered effect of polyamines on expression of virulence factors, including the T3SS**

Response: As the reviewer suggested, we have included the previous studies describing links between host polyamines and bacterial infections in the Introduction section in the revised manuscript, as follows: On the other hand, *S*Tm infection leads to elevated expression of arginase (Arg-1 and Arg-2) [26], which might be a favorable condition for polyamine production. Furthermore, polyamines such as PUT and SPD contribute to *S*Tm virulence by activating the expression of T3SS-1 and T3SS-2 [25] or by inducing a stress response towards reactive oxidative and reactive nitrogen species [23]. Similarly, in *Pseudomonas aeruginosa*, SPD has been shown to modulate T3SS expression [27, 28]. Very recently, it has become clear that SPD is a key factor in *S*Tm pathogenesis by participating in the early stages of infection, including the adhesion and invasion of *S*Tm into host cells [29]. (Lines 77-85 of Pages 3-4 in the revised manuscript)

2. The specific lack of needle formation at low polyamine levels is a central point of the study. In the light of the effect of polyamines on expression levels of many T3SS components, the expression levels of SPI-1 and SPI-2 components, especially the needle subunit, should be tested under the conditions used for the experiments. This would also help to determine the reason for the discrepancies with earlier studies (ref. 23 and Nair et al., see previous point).

o Relatedly, can the authors discriminate between assembly of the needle and secretion of the needle subunit? If the effect is on the secretion, is it possible that an energy deficiency, caused by lack of polyamines and also influencing groth (see Fig. 1) is the cause?

Response: We thank the reviewer very much for the helpful suggestion. We acknowledge that data obtained from this suggestion will help our conclusion regarding the discrepancies with earlier studies showing that polyamines influence the transcriptional activity of T3SS genes. As suggested, we have investigated the expression levels of the needle subunits. The expression levels of the needle subunits PrgI of T3SS-1 and SsaG of T3SS-2 in the polyamine mutant were equivalent to those in the WT strain. Although earlier studies showed that polyamines positively regulate the expression of T3SS-1 and T3SS-2 genes (Jelsbak et al., PloS One 2012, PMID: 22558361; Nair et al., Microbiol Res 2024, PMID: 38232495), our data showed that the impaired activation of T3SS in the polyamine mutant (Δ*speABCEDF* Δ*potAB* Δ*potFGHI*) is not attributable to the reduced expression of T3SS proteins. In conclusion, the impaired assembly of a needle subunit leads to reduced T3SS activity in the Δ*speABCEDF* Δ*potAB* Δ*potFGHI* mutant. We have included this information and explanation in the revised manuscript, as follows: We next investigated the expression levels of the T3SS needle subunit in the Δ*speABCEDF* Δ*potAB* Δ*potFGHI*. Initially, gene expression levels were measured by using a β-galactosidase assay. The HilA positively regulates the *prgHIJK-orgABC* operon, including the *prgI* encoding the needle subunit of T3SS-1 (S8A Fig). Expression levels of *prgH* were decreased in the Δ*hilA* compared to the WT, whereas the *prgH* levels in the Δ*speABCEDF* Δ*potAB* Δ*potFGHI* were similar to those in the WT (S8B Fig). Thus, we next examined the protein expression of the needle subunit. To this end, a plasmid expressing an HA-tagged T3SS protein was constructed and then transformed into the *S*Tm strains. Expression of PrgH-2HA and PrgI-2HA from the plasmids depended on the presence of *hilA*, whereas the protein expression levels of both proteins in the Δ*speABCEDF* Δ*potAB* Δ*potFGHI* were equivalent to those in the WT (S8C and S8D Figs). Likewise, the protein expression levels of SsaG-2HA, the needle subunit of T3SS-

2, were reduced in the Δ*ssrB*, whereas the Δ*speABCEDF* Δ*potAB* Δ*potFGHI* produced the SsaG-2HA at the similar levels to the WT (S8E Fig). These results suggest that the incomplete needle complex of T3SS machinery found in the Δ*speABCEDF* Δ*potAB* Δ*potFGHI* is not attributed to decreased expression of the needle subunit, but rather raises the possibility of impaired assembly of the needle subunit. (Supplemental Figure 8 and Lines 464-480 of Page 18 in the revised manuscript)

It is quite challenging to detect the secreted needle subunits. In contrast, the use of specific antibodies against needle subunits might allow their detection in Western blot analysis. Unfortunately, however, we presently are unable to use the antibodies in our laboratory. On the other hand, given our data that the substrates for T3SS (translocase and effector) are secreted into culture supernatant in Δ*speABCEDF* Δ*potAB* Δ*potFGHI*, it is reasonable to speculate that the needle assembly defect might not be due to an energy deficiency. The reviewer has raised an important question for deciphering the molecular mechanism by which spermidine facilitates the needle assembly. Therefore, we would like to add this topic to our future research. Again, we appreciate the valuable direction.

3. It is rather surprising that the authors observe secretion of middle and late T3SS substrates in the absence of T3SS needles. It was shown that in the absence of the needle, the T3SS secretin adopts a closed state, most likely sealing the T3SS (Hu et al, DOI 10.1038/s41564-019-0545-z). Similarly, strains lacking the needle subunit do not export any T3SS substrate in several bacteria where this was tested. To better understand this unexpected phenotype, which is central to the manuscript, strains lacking the needle component should be used as a control in the respective experiments.

- **Relatedly, is it possible that both strains used form complete T3SS, but lose the needle in lower polyamine conditions, either over time or during the purification procedure? In either case, this central aspect (Fig. 4A) should be supported by bigger fields of view (perhaps as Suppl. Data) and quantification of purified needle complexes with and without needle in both strains.**

Response: We agree with the reviewer that it is quite surprising that the polyamine biosynthesis/transport-deficient *Salmonella* mutant lacking the T3SS needle can secrete middle and late T3SS substrates. Therefore, as the reviewer suggested, we conducted additional experiments with the *prgI Salmonella* mutant, which lacks the needle subunit. As shown in Figure R5 below, we confirmed that the prgI mutant has less invasiveness into HeLa cells compared to the WT. Furthermore, as shown in Figure R6 below, we

found that this mutant strain fails to translocate the T3SS-1 effector into HeLa cells. Finally, we purified the needle complex of T3SS-1 from the *prgI* mutant. Similar to the case with the polyamine biosynthesis/transport-deficient mutant (Δ*speABCEDF* Δ*potAB* Δ*potFGHI*), the needle complex purified from the *prgI* mutant lacks the needle. We added this information in Supplemental Figure 7 of the revised manuscript. Furthermore, we investigated the proportion of needle complexes with needles from *Salmonella* strains grown in LB supplemented with spermidine or without. As shown in Supplemental Figure 7 of the revised manuscript, the addition of spermidine into the culture medium had no effect on needle assembly.

How is spermidine involved in the needle assembly? We still cannot answer this important question. However, one may consider at least two alternative scenarios: (i) "Direct involvement". Spermidine is involved in the process of assembly of the needle subunit. For example, spermidine might interact with the needle subunit, PrgI. (ii) "Indirect involvement". The polyamine biosynthesis/transport-deficient mutant has pleiotropic effects, for example, very attenuated outer membrane integrity. Thus, in this mutant, the needle is assembled, but may fall out of the basal body during bacterial growth. Namely, spermidine might interact with as-yet-unidentified proteins, leading to the needle assembly. Therefore, we propose that future research should focus on this topic to decipher these steps. Thus, we added this information to the revised manuscript, as follows: Furthermore, we investigated the proportion of needle complexes with needles from WT, the needle-subunit-deficient mutant Δ*prgI*, and Δ*speABCEDF* Δ*potAB* Δ*potFGHI* grown in LB supplemented with SPD or without it. Approximately 46% of purified needle complexes from WT grown in LB had the needle (Figs S7A and S7B). In contrast, the Δ*prgI* and Δ*speABCEDF* Δ*potAB* Δ*potFGHI* grown in LB had no needles (Figs S7A, S7D and S7F). The addition of SPD to the culture medium had no effect on needle assembly in any of the tested strains (Figs S7A, S7C, S7E and S7G). (Supplemental Figure 7 and Lines 440-447 of Page 17 in the revised manuscript); How may polyamines facilitate needle assembly? Polyamines are ubiquitous in nature and mammalian cells at high levels (greater than millimolar concentrations), and it is notable that polyamines can interact with proteins by serving as counterions to anionic targets. Indeed, SPD and SPM play regulatory roles in microtubule assembly in cells by controlling the transition of microtubule structures between straight and curved, leading to the formation of bundles [48, 49]. Moreover, it is shown that polyamines control the assembly of neuronal receptors while also acting a gatekeeper [50-53]. Based on the previous observations, it is reasonable to speculate that SPD may interact with the needle subunits through ionic binding, thereby facilitating the assembly. Moreover, the low

isoelectronic point values of the needle subunits, PrgI and SsaG, might facilitate the interaction with polyamines that possess a cationic nature. Furthermore, one may consider an alternative scenario: "Indirect involvement". The Δ*speABCEDF* Δ*potAB* Δ*potFGHI* has pleiotropic effects [23, 25, 29]. Thus, in this mutant, the needle is assembled, but may fall out of the basal body during bacterial growth. Namely, spermidine might interact with as-yet-unidentified proteins, leading to the needle assembly. (Lines 698-712 of Pages 26-27 in the revised manuscript)

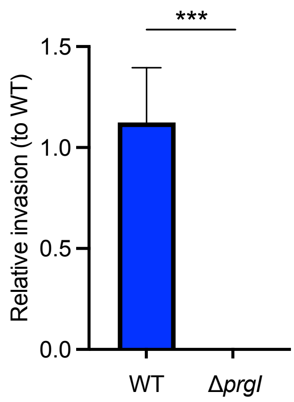

Figure R5. Δ*prgI* mutant shows decreased invasiveness into HeLa cells. HeLa cells were infected with the indicated *S*Tm strains, and the number of invading bacteria was determined by a gentamicin protection assay. Invasion relative to that of the WT was demonstrated across at least three independent experiments. Bars represent means ± SD. ***$P < 0.001$; Mann–Whitney U test.

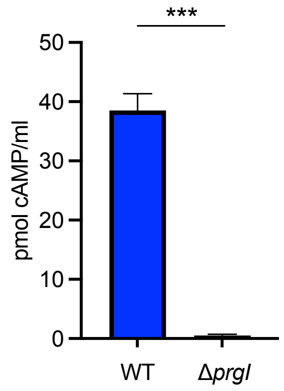

Figure R6. Δ*prgI* mutant fails to translocate the T3SS-1 effector into HeLa cells. Translocation of the SopD-CyaA fusion protein into HeLa cells. Monolayers of HeLa cells were infected with the WT and ΔprgI expressing the SopD-CyaA fusion protein for

2 hours. Bars represent means ± SD from three independent experiments. $^{***}P < 0.001$; Mann–Whitney U test.

4. Overexpression of potAB and addition of polyamines much more than complements the effect of the used deletions in many experiments (e.g, Fig. 3G, S3D, S3E, S3F, S3J, S4E). This implies that bacterial access to polyamines is not only required, but actually limiting for infection. It also raises the question why bacteria don't express higher levels of potAB in infection settings. It would be highly relevant and interesting to expand on this finding.

Alternatively, it might just mean that access to polyamines leads to increased bacterial growth, which then leads to stronger downstream effects. Growth of the motAB overexpression strain should therefore be tested (like in Fig. 1C-F).

Response: This is a very interesting thought. As described above (please see our response to comment #3 of reviewer #1 above), the PotABCD transporter plays a role in cell invasion distinct from polyamine uptake. A study regarding the unknown function of PotABCD and potAB expression for infection settings will be a promising topic for future work. Again, we would like to thank the reviewer for the valuable suggestions.

Following the reviewer's suggestion, we examined the growth of a *Salmonella* strain overexpressing *potAB* in culture with or without polyamines. Results from this experiment showed that the PotABCD transporter contributes to bacterial growth through the uptake of exogenous polyamines. This information and an explanation have been added as Supplemental Figure 4 of the revised manuscript, as follows: Previous results (Figs. S3D and S3G) have shown that overexpression of the PotABCD transporter from the plasmid pMW-*potAB* has much more than a complementary effect, raising the possibility that the PotABCD transporter facilitates bacterial growth independently of SPD uptake. Thus, we have examined the growth of the Δ*speABCEDF* Δ*potAB* Δ*potFGHI* harboring pMW-*potAB* in M9 with or without SPD. In M9 without SPD, the Δ*speABCEDF* Δ*potAB* Δ*potFGHI* harboring pMW-*potAB* exhibited retarded growth compared to the WT (S4 Fig). In contrast, SPD supplementation led to enhanced growth on the Δ*speABCEDF* Δ*potAB* Δ*potFGHI* harboring pMW-*potAB*. These results indicate that expression of the PotABCD transporter contributes to bacterial growth through the uptake of exogenous SPD. (Lines 322-331 of Pages 12-13 in the revised manuscript)

5. Lines 493/494, "These results clearly indicate the causal link between increased SPD levels and enhanced Salmonella pathogenesis." - isn't it equally

possible that increased levels of polyamines exert a stress on the host cells, which makes them more susceptible to infection? This would be an indirect, rather than a causal link.

Response: We apologize if our wording was misleading. We rephrased the sentence as follows: These results clearly indicate a possible link between increased SPD levels and enhanced *Salmonella* pathogenesis. (Lines 627-628 of Page 24 in the revised manuscript)

6. In the results part, results on SPI-1 and SPI-2 need to be more clearly separated. They are two different systems used at very different circumstances. Currently, it is easy to overlook which system the authors discuss in various parts, e.g. in Fig. 3 and Fig. 4.

Response: We agree with the reviewer that our presentation, especially Figure 3 in the original manuscript, was complicated. According to the reviewer's suggestion, we rearranged the figure in the revised manuscript. Please see our response to the first comment of reviewer #1 above.

Minor points:

1. The common Sct T3SS nomenclature (DOI 10.1128/mmbr.62.2.379-433.1998 and 10.1007/82_2020_210) should be used at least in parallel for anyone not familiar with the SPI-1/2-specific names of T3SS components.

Response: We thank the reviewer for pointing this out. We agree that using the common nomenclature will be helpful for readers. We therefore included the common Sct nomenclature in the revised manuscript as follows: The pathogenesis of *S*Tm largely depends on two distinct type 3 secretion systems (T3SS-1 and T3SS-2; refer to the Sct common nomenclature [16, 17] in Supplementary Table 1), (Lines 70-72 of Page 3 in the revised manuscript)

2. Can the authors speculate on the different (sometimes opposite) impact of the mutations on the colonization of feces/cecum vs. spleen?

Response: We acknowledge this important point. In the streptomycin model (oral gavage) using streptomycin-pretreated mice, unlike the typhoid model (intraperitoneal

infection), there is a significant induction of mucosal inflammatory responses that also affect systemic immunity, including the spleen. It is therefore plausible to suggest that gut infectivity influences subsequent colonization in the spleen. On the other hand, because the colonization mechanism is quite different between the gut and spleen, we note that the *Salmonella* mutant strain can colonize the gut at similar levels to the WT but impairs colonization in the spleen (for example, the Δ*potAB* Δ*potFGHI* mutant in this study).

3. Why was a HilD overepxression strain used in Fig. 4BC and how do the results look without HilD overexpression?

Response: Indeed, we have observed the presence of the PrgI polymerization-dependent clump only upon overexpression of HilD. This means that maximal T3SS expression is necessary for bacterial clump formation. Although T3SS is activated in cultures without overexpression, it is speculated that this condition is insufficient, as demonstrated in Fig 6C of the revised manuscript.

4. The reduced colonization levels shown in Fig. 6C are strange in this figure and would fit better into Fig. 3.

Response: As requested, we moved the figure to Figure 3A in the revised manuscript.

5. Fig. 6DE, "Transcript levels" (as indicated in the figure legends) are not equivalent to "Expression" (as indicated in the figure itself).

Response: We apologize that our wording was misleading. We corrected the wording to "Transcript levels" in the figure.

6. Line 507 and perhaps elsewhere, "whiskers denote minimum and maximum", but there are no whiskers below the average.

Response: We are very sorry for this mistake, which we corrected in the revised manuscript.

Suggested text changes:

7. Line 201 "double-deficient" instead of "two T3SS-deficient"

Response: We rephrased the text as suggested. (Line 219 of Page 9 in the revised manuscript)

8. Line 368, "a late substrate" instead of "the". While the article is very well-written and easy to understand, the use of articles is slightly unusual in several instances.

Response: Thank you for pointing this out. We rephrased the text. (Line 495 of Page 19 in the revised manuscript)

9. Lines 467/468, not sure what this sentence means.

Response: We apologize if our wording was insufficiently clear. We intended to state that infection with ΔinvG ΔssaV does not lead to increased expression of Arg-1 and Arg-2. Therefore, we rephrased the sentence as follows: The induction of *Arg* gene expression was not observed in infection with Δ*invG* Δ*ssaV* (Fig 8E). (Lines 601-602 of Page 23 in the revised manuscript)

10. Lines 525-526 and others, what is meant by "functional expression of the T3SS"? A more specific expression is needed. Similarly, "assembly of the T3SS machinery" in line 561-562 - which part exactly?

Response: Thank you for pointing this out. We rephrased "functional expression of the T3SS" with "the assembly of T3SS machinery". (Line 661 of Page 25 in the revised manuscript) In addition, "the assembly of the T3SS machinery" was rephrased to "the needle assembly of T3SS". (Lines 696-697 of Page 26 in the revised manuscript)

11. Line 606-607, "plays a critical role" OR "has consequences for".

Response: As requested, we rephrased the text. (Lines 653-654 of Page 25 in the revised manuscript)

Reviewer #3:

In this manuscript the authors investigate how the polyamines putrescine and

spermidine contribute to *Salmonella* infection of the host. They show that a polyamine biosynthesis mutant and a polyamine transport mutants (spe pot) have slower growth in minimal medium. In addition, they show that the spe pot mutants have a defective production of the T3SS-1 secretion apparatus resulting in reduced invasion in vitro and in vivo. Although several observations confirm previous observations (eg. Polyamines increase Salmonella invasion into host cells in vivo and in vitro, polyamine uptake mutants have a defect in growth, PMID: 31991172, PMID: 27041598, PMID: 22558361, PMID: 38232495) this is a well-written manuscript shedding new light on the role of polyamines for Salmonella pathogenesis.
However, there are several points which remain unclear:

Response: We appreciate the positive comment from reviewer #3 and the reviewer's thoughtful consideration of this issue. Please see our responses to the reviewer's comments below.

1. In Figure 2: the authors conclude that polyamine uptake contributes to salmonella colonization of the spleen. However, they also show in Figure 1 that exogenous polyamine corrects the defective growth of the spe pot uptake mutants. Either there is another polyamine uptake mechanism (but then the in vivo conclusion are questionable) or there is an activity of the polyamines apart from their intracellular activity.

Response: Thank you for pointing this out. In our initial observation, it was indeed surprising that polyamine supplementation reverses the defective growth of the spe pot uptake mutants (Figure 1 in the original manuscript). This raises the possibility that, besides the PotABCD and PotFGHI transporters, *Salmonella* possesses at least one alternative transporter. This also implies that the maintenance of intracellular polyamine levels plays a role in bacterial growth, as it is speculated that PotABCD and PotFGHI are major transporters in *Salmonella*, as earlier studies have demonstrated in *E. coli*. Therefore, we speculate that polyamine supplementation allows the spe pot uptake mutants to maintain intracellular levels of polyamines similar to those of the WT strain. On the other hand, we agree with the reviewer that the data also indicate the possibility that polyamine has other effects beyond intercellular functions. To this end, we thus have investigated the intracellular polyamine levels in *Salmonella* strains grown in media supplemented with polyamines or not supplemented. The results are presented in Figure

R1 above (please see Figure R1 in our response to comment #1 from reviewer #1). As evident from these data, the spe pot uptake mutant imports polyamines into the intracellular compartment even in the absence of the PotABCD and PotFGHI transporters. This supports the conclusion that *Salmonella* possesses another polyamine uptake mechanism apart from the PotABCD- and PotFGHI-mediated ones. We added this additional information and an explanation in the revised manuscript, as follows: To explore the possibility, we investigated the intracellular contents of polyamines of *S*Tm strains grown in M9 supplemented with SPD or without supplementation. In M9 without SPD, intracellular SPD was detected in the WT but not Δ*speABCEDF* or Δ*speABCEDF* Δ*potAB* Δ*potFGHI* (Fig 1E). In contrast, intracellular SPD was detected at similar levels in all tested strains grown in M9 containing SPD. These results indicate that Δ*speABCEDF* Δ*potAB* Δ*potFGHI* can import exogenous polyamines through an as-yet-unidentified transporter. (Lines 119-125 of Page 5 in the revised manuscript)

2. Figure 3: are the uptake mutants potAB or potAB potFGHI also defective for invasion? The complementation experiments would suggest this but this experiment could answer whether potAB or potFGHI have additional functions in bacterial invasion apart from polyamine uptake.

Response: We appreciate this excellent suggestion. Please see our response to comment #3 of reviewer #1 above. As that reviewer indicated, the potAB potFGHI mutant is less invasive than the WT (Figure R3 in our response to comment #3 from reviewer #1 above). Furthermore, we found that the transformation of a plasmid expressing PotAB restores the invasiveness of the potAB potFGHI mutant. It should be noted that polyamine supplementation has no effects on the defect, indicating that because the potAB potFGHI mutant can synthesize polyamines, the defect is not attributable to the reduction in intracellular polyamine levels. Next, we investigated the T3SS-1 activity of the potAB potFGHI mutant in a mouse model. As can be seen in Figure R4 and in our response to comment #3 from reviewer #1 above, the T3SS-1–dependent gut inflammation levels of the potAB potFGHI mutant were identical to those of the WT. Collectively, these new data indicate the possibility that beyond polyamine transport, the PotABCD transporter itself contributes to bacterial invasion in a manner independent of polyamine homeostasis. We added this information to the revised manuscript, as follows: On the other hand, the invasiveness of Δ*potAB* Δ*potFGHI* into HeLa cells was impaired (S3E Fig). The reduced invasion capacity was partly reversed by introducing a plasmid encoding *potAB* but not *potFGHI*. In contrast, unlike the Δ*speABCEDF* Δ*potAB* Δ*potFGHI*, SPD

supplementation had no complementary effect on the invasion defects of the Δ*potAB* Δ*potFGHI* (S3E Fig), indicating that the PotABCD transporter contributes to the cell invasion of *S*Tm in a manner independent of polyamine uptake. (Lines 298-304 of Page 12 in the revised manuscript)

3. Also as exogenous spermidine helps in bacterial invasion of the uptake mutant, this hints at an additional mechanism.

Response: Please see our response to comment #2 above. Spermidine supplementation reverses the reduction in invasion of the speABCEDF potAB potFGHI mutant but not the potAB potFGHI mutant, indicating that uptake-dependent polyamine homeostasis and the PotABCD transporter contribute to bacterial invasion independently. The new data showed that the PotABCD transporter has dual roles in the cell invasion of *S*Tm. Again, we would like to thank the reviewer for the valuable suggestion.

4. Is invasion of the spe pot mutant into Raw cells different from invasion of wt bacteria?

Response: Thank you for pointing this out. In response, we added the following to the revised manuscript: Similar to the results of invasion into HeLa cells, we found that the invasiveness of Δ*speABCEDF* Δ*potAB* Δ*potFGHI* into RAW264.7 cells was lower compared to the WT, a deficit that was restored by transformation of a plasmid encoding *potAB* (S6C Fig). (Lines 380-383 of Page 15 in the revised manuscript)

5. Fig 3E: as a control to exclude and possible T3SS1-unrelated effects of spermidine it would be important to know if spermidine also affects SopD translocation in the invG mutant.

Response: We acknowledge this important comment. According to the reviewer's suggestion, we conducted an additional experiment to investigate whether the invG mutant grown in culture medium supplemented with spermidine injects SopD into host cells. As expected, the spermidine addition-dependent enhancement of the effector translocation depended on the functionality of T3SS-1. We included this information in Supplemental Figure 3J and in the following sentences in the revised manuscript: SPD supplementation had no effect on the Δ*invG*, indicating that SPD-dependent enhancement of effector translocation depends on the functionality of T3SS-1 (S3J Fig). (Lines 339-

340 of Page 13 in the revised manuscript)

6. Fig S4: spermidine increases spe pot and wt mediated cytotoxicity. The authors conclude that spermidine increased T3SS2 dependent cytotoxicity. In this case, ssaV dependent cytotoxicity should not be affected by spermidine treatment. This should be tested.

Response: Like comment #5 by reviewer #3, this comment is important. According to the reviewer's suggestion, we investigated the effect of spermidine supplementation on ssaV-dependent cytotoxicity. Spermidine supplementation had no effect on T3SS2-dependent cytotoxicity. We thus added the following information in Supplemental Figure 6F: The effect of SPD supplementation depended on T3SS-2-dependent cytotoxicity (S6F Fig). (Lines 395-396 of Page 15 in the revised manuscript)

7. For the effect of exogenous added spermidine on intracellular Salmonella: how is spermidine delivered into the SCV? Can this be tested? This should be discussed.

Response: We would like to thank the reviewer for raising this important question. We also acknowledge that this point is a very promising topic because delivery of polyamines to SCV might become a target for regulating *Salmonella* infection. In general, it is reasonable to assume that host nutrients, including polyamines, are delivered to the phagolysosome via fusion with phagocytic and endocytic vesicles (McConville et al., Trends Parasitol 2007, PMID: 17606406; McConville and Naderer, Annu Rev Microbiol 2011, PMID: 21721937). However, it remains largely unknown how polyamine is transported into the vacuole.

The levels of polyamines such as putrescine and spermidine are very low in macrophages under physiological conditions (Boitz et al., Infect Immun 2016, PMID: 27795357; Minois et al., Aging 2011, PMID: 21869457; Pegg, IUBMB Life 2009, PMID: 19603518). In contrast, supplementing exogenous polyamines through foods or drinking water rich in polyamines can transiently increase their levels within infected cells, as evidenced by previous studies (Olenyik et al., Mol Biochem Parasitol 2011, PMID: 21182873; Gaur et al., J Immunol 2007, PMID: 18056391) and our data (Supplemental Figure 12 in the revised manuscript). So far, several putative mammalian polyamine transporters have been identified. However, a comprehensive understanding of the process is still lacking. Thus, this will be an important topic for future work. Therefore,

we included the following sentences in the revised manuscript: Based on our results, it is reasonable to assume that inhibiting polyamine import into the vacuole containing *Salmonella* (referred to as SCV, *Salmonella*-containing vacuole) might be a promising therapeutic target. To date, several putative mammalian polyamine transporters have been identified [82-88]. However, a comprehensive understanding of the process is still lacking. Therefore, a study that sheds light on the molecular mechanism by which polyamines are delivered into the SCV will be promising and necessary. (Lines 768-774 of Page 29 in the revised manuscript)

8. Fig 4A: does exogenous spermidine addition correct the formation of the entire needle complexes?

Response: Thank you for pointing this out. Please see our response to comment #3 from reviewer #2 above.

9. Fig 4B: why is SPI-1 not induced under these growth conditions and why is overexpression of HilD needed?

Response: We apologize if the figure in our original manuscript was unclear. Indeed, these growth conditions in Fig. 6B of the revised manuscript (Fig. 4B of the original manuscript) are sufficient to induce SPI-1 activity. On the other hand, as shown in Fig. 6C of the revised manuscript, bacterial clumping requires the maximum expression of SPI-1 by overexpression of HilD. In addition, the overexpression of HilD can ensure homogenous expression of SPI-1.

10. Fig 6: is the increase of polyamines a general feature during inflammation? Eg is the same effect seen in DSS colitis? This should be discussed.

Response: Thank you very much for raising this concern. We fully agree with this important comment. Therefore, we determined the polyamine levels in feces and spleen of the DSS model mouse. As expected, the polyamine levels were increased in the DSS model mouse compared to the untreated mouse. Therefore, we included this new finding in Supplemental Figs 10A and 10B and in the following sentences in the revised manuscript: We next asked whether the elevation of polyamine levels in the mice infected with *S*Tm is part of the host inflammatory response. To this end, polyamine contents in the feces and spleen from a mouse model of dextran sulfate sodium (DSS)-induced colitis

were measured. As expected, the DSS model mouse showed higher polyamine levels in feces and spleen compared to the untreated mouse (Figs S10A and S10B), lending support to the hypothesis that host inflammation facilitates the production of polyamines. (Lines 583-588 of Page 22 in the revised manuscript)

11. It was recently shown that spe pot mutants are also defective for production of siiE and flagella which also explains the defective uptake into host cells and diminished virulence in vivo (PMID: 38232495). This needs to be discussed.

Response: Thank you for the suggestion. In response, we included the following in the Introduction and Discussion sections in the revised manuscript: Very recently, it has become clear that SPD is a key factor in *S*Tm pathogenesis by participating in the early stages of infection, including the adhesion and invasion of *S*Tm into host cells [29]. (Lines 83-85 of Page 4 in the revised manuscript); More recently, it has been shown that *Salmonella* utilizes SPD for the expression of the genes involved in the giant adhesin SiiE, flagella, and T3SS-1, contributing to the early stages of pathogenesis [29]. (Lines 650-652 of Page 25 in the revised manuscript)

12. Statistics: Can the authors please explain the choice of their statistical tests. For example, in Fig 3 CD anova was used but in fig 3E student t test was used although more than two groups were compared; in Fig 1GH: student T test was used but more than two groups were compared? Which post-hoc tests were used after ANOVAs?

Response: We sincerely apologize that our analyses have been misleading. In response to the reviewer's concern, we employed appropriate statical analyses in the revised manuscript.
