# Supplementary material for: Salmonella Typhimurium exploits host polyamines for assembly of the type 3 secretion machinery
Source: PLoS Biol. 2024 Aug 5;22(8):e3002731. doi: 10.1371/journal.pbio.3002731 (PMC11299824; doi:10.1371/journal.pbio.3002731)
Supplement: Attachment — Submitted filename: Response v2-1.pdf [file pbio.3002731.s021.pdf]

Point-by-point responses to Reviewers' comments
Manuscript ID#: PBIOLOGY-D-24-00105R2

Dear colleagues,

Thank you for the assessment on our revised manuscript, PBIOLOGY-D-24-00105R2, entitled "*Salmonella* exploits host polyamines for assembly of the type 3 secretion machinery". In response to the reviewer comments, we conducted several additional experiments and have improved the manuscript. The text changes in the revised manuscript are highlighted in red. Reviewer comments appear in bold, followed by our point-by-point responses.

We believe that the below described additions and adjustments satisfy the remaining concerns of reviewer #1 and reviewer #2. We would like to thank all three reviewers for their valuable input and look much forward to your response.

With best regards,
The Authors

Reviewer #1:
I thank the authors for their extensive response and considerations related to the comments given by the reviewers. From my point of view the comments were intensively addressed, discussed, and where indicated translated into an improved outline and wording of the manuscript.

I only have one further comment: Do the authors have any explanation on why spermidine supplementation doesn't restore T3SS-1 needle assembly in the ΔspeABCEDF ΔpotAB ΔpotFGHI mutant (Fig S7A-F)? This should be addressed and discussed, since polyamines effect on T3SS assembly is the central/novel result of the manuscript.

Response: We are happy to hear that reviewer #1 finds our revisions compelling. Furthermore, thank you very much for pointing this out. We agree to the reviewer that the regulatory role of polyamines in T3SS assembly is the central/novel results of our manuscript.

In line with previous observations, requirement of the needle assembly for secretion of the T3SS substrates was demonstrated by our results that the needle subunit-deficient strain (prgI mutant) cannot secrete any substrates. In contrast, the secretion was observed in ΔspeABCEDF ΔpotAB ΔpotFGHI strain that fails the needle assembly. On the basis of these results, it is reasonable to speculate that in ΔspeABCEDF ΔpotAB ΔpotFGHI strain, the needle is transiently assembled, but not stable for any reason. Given our data that addition of spermidine into the culture media restores translocation of the T3SS effector in ΔspeABCEDF ΔpotAB ΔpotFGHI strain, we also speculate that the spermidine supplementation may transiently facilitate the needle assembly, but was not sufficient for the stable expression. Therefore, we added the information and explanation to page 27 of the revised manuscript as follows: Given our data that the Δ*speABCEDF* Δ*potAB* Δ*potFGHI* can secrete T3SS effectors into culture media, it might be reasonable to speculate that the needle is transiently assembled in this mutant because the needle-subunit-deficient mutant Δ*prgI* cannot secrete any T3SS effectors. On the other hand, we further speculate that the needle assembly is unstable in the Δ*speABCEDF* Δ*potAB* Δ*potFGHI* for any reason. In this model, the assembled needle easily falls out of the basal body of T3SS. In contrast, our results showed that addition of SPD into the culture media restore the translocation of T3SS effector in the Δ*speABCEDF* Δ*potAB* Δ*potFGHI*. Therefore, the SPD supplementation may transiently facilitate assembly of the needle, but was not sufficient for stable expression of the needle, as shown by the results that addition of SPD into the culture media does not restore the needle assembly in the Δ*speABCEDF* Δ*potAB* Δ*potFGHI*. (Lines 709-720 of Page 27 in the revised manuscript)

Reviewer #2:

The authors have addressed most of the requests and clarified many points, which further improves the manuscript.

However, in response the request to test the secretion of middle and late T3SS substrates in absence of the T3SS needle subunit, the authors present data that absence of needles decreases invasiveness and effector translocation, but not on secretion of middle and late substrates.

These experiments should be included and interpreted, given that the surprising finding that the needle-less ΔspeABCEDF ΔpotAB ΔpotFGHI strain secrete middle and late substrates is central to the manuscript.

Response: We apologize that we did not address the reviewer's concern adequately in

our first revision. We performed the suggested experiment with a prgI mutant lacking the needle subunits. In line with previous reports, we confirmed the requirement of needle assembly for secretion of the T3SS substrates. Thus, given our results that ΔspeABCEDF ΔpotAB ΔpotFGHI strain secretes the T3SS substrates, it is reasonable to speculate that in this mutant, the needle subunits are transiently assembled, allowing for the substrate secretion. On the other hand, we also speculate that the needle might be unstable for any reason, which disables the translocation of T3SS substrates in ΔspeABCEDF ΔpotAB ΔpotFGHI. Thus, on page 20 of the revised manuscript we report the information as follows: To clarify the requirement of needle assembly for secretion of T3SS substrates, we asked whether the Δ*prgI*, which lacks the needle subunit, can secrete a middle substrate (SipB) and a late substrate (SopD) into the culture media. The WT and Δ*speABCEDF* Δ*potAB* Δ*potFGHI* secreted SipB into the culture media at the similar levels (S9A Fig). In contrast, the secretion of SipB was not observed in the Δ*invG* and Δ*prgI*. Similar results were observed in the secretion of SopD-2HA (S9B Fig). Furthermore, we confirmed that the Δ*prgI* failed the substrate translocation (SopD-CyaA) and invasion into HeLa cells (Figs S9C and S9D). These results indicate that assembly of the needle subunits is required for secretion of the T3SS substrates. Furthermore, the results raise the possibility that the Δ*speABCEDF* Δ*potAB* Δ*potFGHI* can transiently express the needle for the secretion of T3SS substrates. (Lines 520-530 of Page 20 in the revised manuscript)

Reviewer #3:

The revised version of this manuscript has answered my concerns and I think it is now ready to be published.

Response: We appreciate reviewer #3 for constructive input on the original version of the manuscript. We are happy to learn that the reviewer finds our revisions thorough and complete.
